# The Length of *N*-Glycans of Recombinant H5N1 Hemagglutinin Influences the Oligomerization and Immunogenicity of Vaccine Antigen

**DOI:** 10.3389/fimmu.2017.00444

**Published:** 2017-04-20

**Authors:** Agnieszka Katarzyna Macioła, Maria Anna Pietrzak, Piotr Kosson, Mariusz Czarnocki-Cieciura, Krzysztof Śmietanka, Zenon Minta, Edyta Kopera

**Affiliations:** ^1^Institute of Biochemistry and Biophysics, Polish Academy of Sciences, Warsaw, Poland; ^2^Mossakowski Medical Research Centre, Polish Academy of Sciences, Warsaw, Poland; ^3^Laboratory of Protein Structure, International Institute of Molecular and Cell Biology, Warsaw, Poland; ^4^Laboratory of RNA Biology and Functional Genomics, Institute of Biochemistry and Biophysics, Polish Academy of Science, Warsaw, Poland; ^5^Department of Poultry Diseases, National Veterinary Research Institute, Puławy, Poland

**Keywords:** avian influenza, H5N1, *N*-glycosylation, high-mannose glycosylation, low-mannose glycosylation, recombinant hemagglutinin, *Pichia pastoris*

## Abstract

Hemagglutinin glycoprotein (HA) is a principle influenza vaccine antigen. Recombinant HA-based vaccines become a potential alternative for traditional approach. Complexity and variation of HA *N*-glycosylation are considered as the important factors for the vaccine design. The number and location of glycan moieties in the HA molecule are also crucial. Therefore, we decided to study the effect of *N*-glycosylation pattern on the H5 antigen structure and its ability to induce immunological response. We also decided to change neither the number nor the position of the HA glycosylation sites but only the glycan length. Two variants of the H5 antigen with high mannose glycosylation (H5_hm_) and with low-mannose glycosylation (H5_Man5_) were prepared utilizing different *Pichia* strains. Our structural studies demonstrated that only the highly glycosylated H5 antigen formed high molecular weight oligomers similar to viral particles. Further, the H5_hm_ was much more immunogenic for mice than H5_Man5_. In summary, our results suggest that high mannose glycosylation of vaccine antigen is superior to the low glycosylation pattern. Our findings have strong implications for the recombinant HA-based influenza vaccine design.

## Introduction

Traditional manufacturing of influenza vaccines involves using living viruses and presents unique technical and biosafety challenges. Serious limitations of the egg-based method forced researchers for a new solution in the area of influenza vaccine development. Alternative methods are now being explored. A relevant characteristic of the influenza virus is its capacity to change constantly, which is caused by mutations in the genes encoding two proteins: hemagglutinin (HA) and neuraminidase (1). Those changes are usually activated by two mechanisms: antigenic shift and antigenic drift. Both of them may cause the formation of a highly pathogenic virus. The capacity of influenza virus to change constantly is a great challenge for the vaccine production and the main reason why the composition of an influenza vaccine must be reviewed and updated each year.

Recombinant technology enables to obtain the protein product within several weeks, thus the time scale for the influenza vaccine production is much more attractive. As the infection-blocking antibodies are directed mainly against the hemagglutinin protein, a dynamic development in a HA-based subunit vaccine research is currently being observed. This type of vaccines contain recombinant HA protein obtained by means of genetic engineering in different expression systems (2–5). Hemagglutinin is a homotrimeric protein anchored on the surface of the virus. Each monomer consists of two subunits: HA1 and HA2, linked by a disulfide bond. The protein undergoes N-linked glycosylation and its final molecular weight (MW) is approximately 80 kDA.

Glycosylation is the most common post-translation modification by which oligosaccharides are covalently attached to either the side chain of asparagine (N-linked) or serine/threonine (O-linked). In the recent years, glycosylation, especially the N-linked one, has become an area of intensive study due to its ability to impact virus biology (6, 7). It was shown that the *N*-glycosylation of the influenza hemagglutinin plays an important role in the life cycle of influenza virus and influences its antigenic fitness. Indeed, oligosaccharides attached to the globular head of HA were shown to modulate virus antigenic properties (8–10) and its receptor binding (11, 12). The oligosaccharides attached to the stem region were suggested to play a critical role in HA cleavage, replication, and pH stability (13–15). The other research groups also showed that the *N*-glycosylation is required for the efficient folding and oligomerization of HA protein (7, 16–20).

Because the complexity and variation of hemagglutinin glycosylation are considered as the important factors for the influenza vaccine design, it was of interest to study whether the low-mannose glycosylation pattern improves immunogenicity of the H5 influenza antigen. In this study, we used a glycoengineered *Pichia pastoris* strain, which is capable to modify glycoproteins with Man_5_GlcNAc_2_ *N*-glycans. It was reported that all human cells use this glycan moiety as a foundation to form complex glycans (21). Previously, we showed that immunization with the subunit vaccine based on the extracellular region of H5 hemagglutinin with deletion of the multibasic cleavage site fully protected chickens from lethal infections by the highly pathogenic H5N1 virus (22). We also demonstrated that such an antigen spontaneously oligomerized into spherical structures. For the antigen production, we utilized very simple, low-cost, and efficient yeast expression system. In order to obtain antigen variants with a different *N*-glycosylation pattern, recombinant proteins were produced in KM 71 or GlycoSwitch^®^ *P. pastoris* strain. Thus, we obtained high-mannose (H5_hm_) and low-mannose glycosylated (H5_Man5_) H5 antigens having identical protein backbone and only differing in their N-linked glycans. Since differences in the glycosylation pattern of the recombinant proteins might have an impact on their structures, we first specifically investigated this aspect. Then, we compared the immunological properties of the H5_hm_ and H5_Man5_ antigens *in vivo* applying mice model. Our results are highly relevant to the subject of recombinant influenza vaccine antigens.

## Materials and Methods

### Cloning of H5 Hemagglutinins

The DNA encoding the extracellular domain of HA with deletion of the cleavage site (EpiFluDatabase Accession No. EPI15789) from H5N1 avian influenza virus (A/swan/Poland/305-135V08/2006 clade 2.2.2) was cloned into pJAZs1 vector using *Bsa*I (Thermo Scientific, USA) and pPICZαC using *Cla*I and *Not*I restriction sites. pJAZs1/H5_Man5_ and pPICZαC/H5_hm_ plasmids were linearized with *Pme*I (Thermo Scientific, USA) and used for the electroporation of the SuperMan5 (GlycoSwitch, Biogrammatics, USA) and KM 71 (his4, aox1:ARG4, arg4) (Invitrogen, USA) *P. pastoris* strains. Positive clones were transformed to a fresh YPD agar plate with Zeocine (Invitrogen, USA). The yeast transformants were screened for insertion by PCR with 5′ AOX I and 3′ AOX I primers. Yeast clones with verified inserts were grown as previously described (23). The presence of recombinant proteins both in medium and cells (control) was detected by SDS-PAGE and Western blotting.

### Purification of Recombinant HA Protein

Yeast medium was concentrated using tangential flow filtration (TFF) with 10 kDa cutoff Biomax cassette (Millipore, USA) and diafiltrated with 10 mM Tris pH 7.6 (Buffer A). Samples were centrifuged (14,000 rpm, 5 min, 4°C) followed by injection into HiTrap Q HP column. Proteins were eluted with 10 mM Tris pH 7.6 and 1 M NaCl (Buffer B) using a linear gradient from 10 to 45% of Buffer B at a flow rate of 0.25 ml/min. Fractions containing H5 proteins were collected, pooled, lyophilized, and stored at −20°C. H5 antigens were next loaded on a Superdex 200 10/300 GL column (GE Healthcare, UK), pre-equilibrated with 10 mM Tris pH 7.6 with 200 mM NaCl and the protein elution was monitored at 280 nm. MW standards (Bio-Rad, USA) were used to calibrate the column and to identify the MWs of proteins present in the samples.

### Mass Spectrometry Analysis

H5 antigens were denatured with denaturing buffer at 95°C for 10 min. The reaction mix containing denatured HA protein and 0.125 U of endoglycosidase H (Endo H, New England Biolabs, USA) was incubated at 37°C for 1 h. The non-treated protein sample was used as a control. The protein was analyzed using SDS-PAGE. The gel bands containing deglycosylated polypeptides were excised and analyzed by LC–MS–MS/MS (liquid chromatography coupled to tandem mass spectrometry) as previously described (23). Fragmentation spectra of peptides indicated by Mascot as *N*-glycosylated were manually investigated.

### Size Exclusion Chromatography (SEC)–Multi-Angle Light Scattering (MALS) Analysis

The averaged MW of H5hm and H5Man5 oligomers present in the SEC profile after final purification step was determined by SEC–MALS. To this end, protein samples from selected SEC fractions (100 µl) were reinjected into a Superose 6 Increase column (GE Healthcare) equilibrated with a SEC–MALS buffer (10 mM Tris pH 7.6, 200 mM NaCl) at a 0.5 ml/min flow rate. Elution of the proteins was monitored by three online detectors: UV detector (1,220 Infinity LC System, Agilent Technologies, USA), light scattering detectors (DAWN HELEOS II, Wyatt Technology, USA), and refractive index detector (Optilab T-rEX, Wyatt Technology). Data analysis and MW calculations were performed using the ASTRA 6 software (Wyatt Technology).

### Transmission Electron Microscopy (TEM)

The H5_hm_ protein sample was applied to the clean side of carbon on mica and negatively stained with 2% (w/v) sodium silico tungstate. A grid was then placed on top of the carbon film which was subsequently air dried. Images were taken under low-dose conditions (less than 20 e-/A2) with a T12 FEI electron microscope at 120 kV using an ORIUS SC1000 camera (Gatan, Inc., Pleasanton, CA, USA).

### Hemagglutination Assay

Hemagglutination test was performed according to the standard protocol. Briefly, twofold dilution of the H5 antigens was incubated with 1% chicken erythrocytes for 30 min at room temperature.

### Immunization Experiments

The experimental and control groups consisted of 10 7-week-old Balb/c mice. The animals were kept at a constant temperature of 22–24°C. In the first experiment, mice were immunized with 25 µg of H5_hm_ oligomers, H5_hm_ oligomers and monomers, and H5_Man5_ monomers dissolved in saline solution and supplemented with Alhydrogel (aluminum hydroxide, Gentaur, Germany). 100 µl of vaccine was administered by subcutaneous injection into the neck skin fold. There were three injections (first application of antigen and/or adjuvant + two booster shots) at an interval of 3 weeks between each dose in order to monitor immunological response. In the second experiment, mice were immunized twice with 5 µg of H5_hm_ monomers or oligomers and 25 µg of H5_hm_ monomers. An adequate portion of the protein was dissolved in saline plus adjuvant. Control mice received only a specific adjuvant. Blood samples were taken 14 days after each injection in order to determine the level of antibodies. Sera were stored at −20°C.

### Enzyme-Linked Immunoabsorbant Assay (ELISA)

Collected sera were assayed for antibodies against H5 HA by an ELISA method, using MediSorp plates (Nunc, Denmark) coated with mammalian cell-expressed HA (19-529, ΔRRRKKR, 6xHis-tag at C-terminus, Immune Technology, USA) of H5N1 virus (A/Bar-headed Goose/Qinghai/12/05 H5N1, clade 2.2, 99.61% of aminoacid sequence similarity to A/swan/Poland/305-135V08/2006) diluted in PBS to 3 µg/ml. Sera samples, taken from individual groups at each time point of experiment were pooled, serially diluted in 2% BSA/PBS and applied onto the coated plates (o/n, 4°C). Sera samples from mice immunized with H5 protein were tested in parallel with sera from sham-immunized mice (negative controls). Bound antibodies were subsequently detected with horseradish peroxidase (HRP)-labeled goat antibodies against mouse IgG (γ-chain specific, Sigma-Aldrich, USA) at 1:1,000 dilution in 2% BSA/PBS (1 h, 37°C). TMB was used as a HRP substrate. After incubation for 30 min at room temperature, the reaction was stopped by addition of 0.5 M sulfuric acid. The absorbance was measured at 450 nm (A_450_) with a microplate reader (Synergy HTI; BioTek Instruments, USA). Endpoint titer was defined as the highest dilution producing an A_450_ value fourfold higher than the mean A_450_ value of the control group.

### Hemagglutination Inhibition (HI) Test

Sera samples were heat inactivated at 56°C for 30 min and then were pretreated by kaolin and chicken erythrocytes to avoid a false positive reaction in HI test (24). The pretreated sera (10 µl of sera in serial twofold dilutions) were incubated for 25 min in titration plate with four HA units of the inactivated antigen A/turkey/Poland/35/2007 H5N1, clade 2.2.3 (99.22% of aminoacid sequence similarity to A/swan/Poland/305-135V08/2006). Next, a 1% suspension of chicken erythrocytes was added and incubated for 30 min. The HI titer was assessed as the reciprocal of the highest dilution in which hemagglutination was inhibited.

## Results

### Purification of the H5_hm_ and H5_Man5_ Antigens

**Figure 1:**
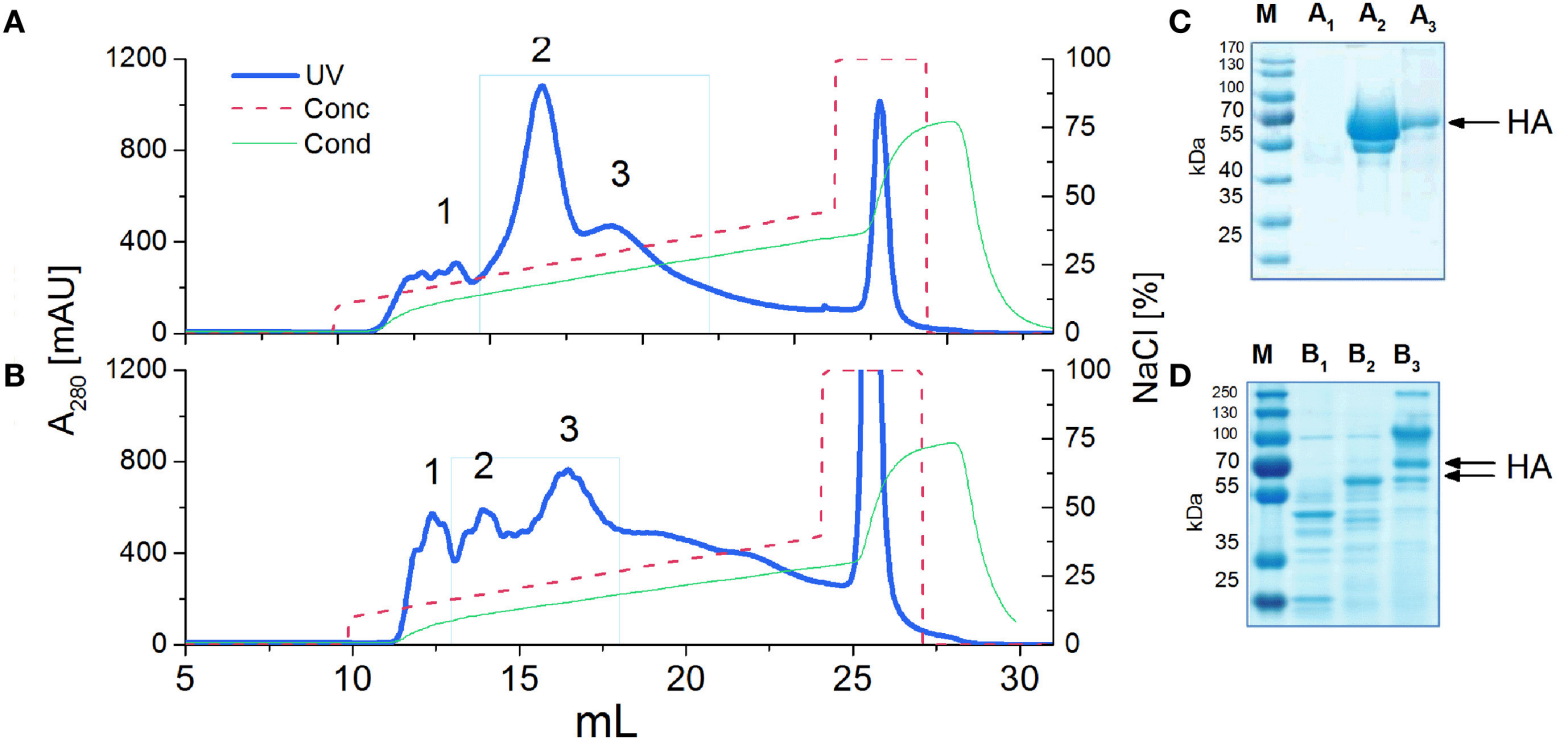
**Ion exchange chromatography (IEC) (A,B) and SDS-PAGE analysis (C,D) of H5 antigens**. IEC chromatograms **(A,B)** present typical purification profiles of H5_hm_ and H5_Man5_ proteins, respectively. Absorbance at 280 nm is shown. HiTrapQ column was pre-equilibrated with 10 mM Tris/HCl pH 7.6 (Buffer A). Proteins were eluted with 10 mM Tris/HCl pH 7.6; 1 M NaCl (Buffer B) applying a linear gradient from 10% B to 45% B at flow rate 0.25 ml/min. Collected fractions (1, 2, 3) were analyzed on 4−12% SDS-PAGE **(C,D)** following Coomassie staining. HA protein was detected in peak no 2 and 3 [**(C)**: line A_2_ and A_3_; **(D)**: line B_2_ and B_3_]. Fractions with H5_hm_ or H5_Man5_ protein were pooled, lyophilized, and stored in −20/−80°C.

The extracellular domain of H5N1 hemagglutinin (residues 17-531, ΔRRRKKR) was selected because it adopted the correct three-dimensional structure required for higher oligomerization. The vaccine based on this HA domain fully protected chickens from lethal infections by the highly pathogenic H5N1 virus (22). This time, in order to obtain the antigens with a native protein sequence, we excluded any affinity tags. Antigen variants with different *N*-glycosylation patterns were produced in the KM 71 or GlycoSwitch^®^ *P. pastoris* strain. The last is engineered to produce proteins with Man_5_GlcNAc_2_ Asn-linked glycans. Finally, we obtained the high-mannose (H5_hm_) and low-mannose glycosylated (H5_Man5_) H5 antigens having identical protein backbone and only differing in their N-linked glycans. In order to increase a sample-to-volume ratio and to simplify the utilization of the ion exchange chromatography (IEC), the yeast medium was concentrated. Various methods were tested, e.g., trichloroacetic acid, ammonium sulfate, dry dialysis using Aquacide and TFF. The last one proved to be the most efficient method (data not shown). Not only it allowed us to concentrate the medium up to 10-fold but also to diafiltrate the protein samples with the IEC buffer. SDS-PAGE analysis after the IEC (Figures 1A,B) showed a high level of purity only for the H5_hm_ protein (Figure 1C). Both recombinant antigens were clearly the major components of the IEC preparations but the purity of H5_Man5_ antigen (Figure 1D) had to be improved during the next purification step applying size exclusion chromatography (see *N*-Glycan-Dependent Structure of the H5_hm_ and H5_Man5_ Antigens). Yield as high as 200 mg of the purified H5_hm_ protein per 1 l was achieved after the IEC procedure. The efficiency of the H5_Man5_ antigen production was rather modest (1.5 mg/l), making the utilization of GlycoSwitch^®^ *P. pastoris* strain as a vaccine platform unattractive.

**Figure 2:**
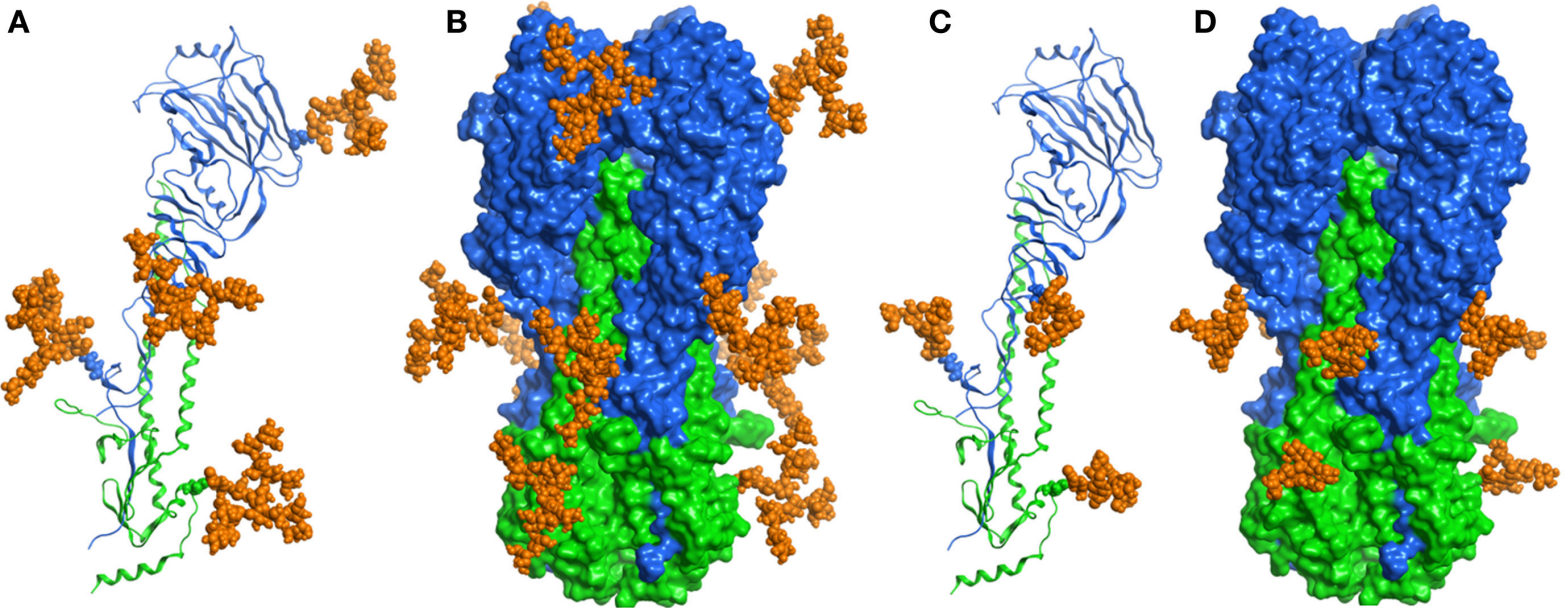
**Structural representations of the molecular models of *N*-glycosylated H5_hm_ (A,B) and H5_Man5_ (C,D) monomers and trimers**. HA1 domain was depicted in blue ribbon representation, HA2 domain—in green one, and oligosaccharides—in orange atomistic ball one. The sugar-bonded asparagines were also depicted in atomistic representations according to their respective domain colors. The models were constructed based on the crystallographic structure of the H5 hemagglutinin (PDB ID code 5E2Y).

In order to examine *N*-glycosylation sites of recombinant HA antigens, we used a standard proteomic procedure for Endo H treated proteins. Mass spectrometry analysis confirmed that four sites in H5_hm_ and three sites in H5_Man5_ are glycosylated (Table 1; Supplementary Material). Three *N*-glycosylated sites (N22, N286, N478) confirmed by LC/MS/MS analysis were common for the H5_hm_ and H5_Man5_ proteins. Additionally N165 was confirmed for the H5_hm_ protein. The structures of glycosylated H5_hm_ and H5_Man5_ monomer and trimer are presented in Figure 2.

**Table 1:** ***N*-glycosylated peptides from the H5_hm_ and H5_Man5_ proteins confirmed by LC/MS/MS analysis**.

Residue	Region	Amino acid sequence	Mass	Antigen
22-35	HA1	**NVT**VTHAQDILEK + HexNAc	1,669.85	H5_hm_ H5_Man5_
163-189	HA1	SY**NNT**NQEDLLVLWGIHHPNDAAEQTR + HexNAc	3,337.56	H5_hm_
278-304	HA1	CQTPIGAI**NSS**MPFHNIHPLTIGECPK + HexNAc	3,221.53	H5_hm_ H5_Man5_
478-491	HA2	**NGT**YDYPQYSEEAR + HexNAc	1,894.78	H5_hm_ H5_Man5_

*N-linked glycosylation sites are bold and underlined. Molecular mass of modified peptides (one HexNAc attached to the asparagine residue) was determined by spectrometer*.

*HexNAc (*N*-acetylhexoseamine)*.

### *N*-Glycan-Dependent Structure of the H5_hm_ and H5_Man5_ Antigens

**Figure 3:**
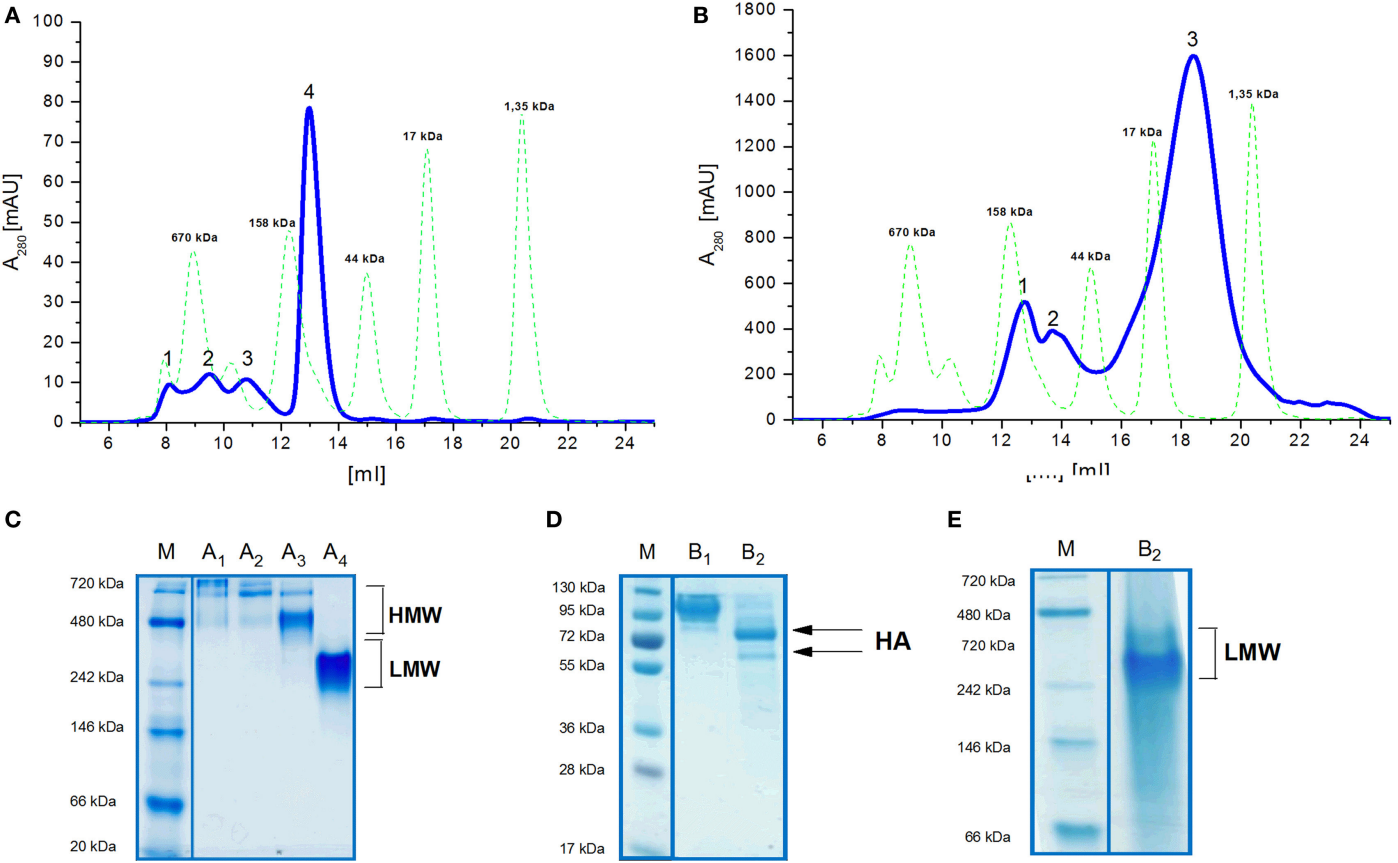
**Size exclusion chromatography (SEC) (A,B) of H5 antigens on Superdex 200 10/300 GL column and Native PAGE analysis (C,E) of SEC fractions**. Chromatograms of the ion exchange chromatography–elution fractions **(A,B)**. Molecular weight (MW) standard is indicated by the green dotted line. Fractions of the H5_hm_ or H5_Man5_ proteins were lyophilized separately and dissolved in water followed by 4−7% Native PAGE **(C,E)**. SEC fractions of the H5_Man5_ protein were additionally analyzed on 4−12% SDS-PAGE **(D)** following Coomassie staining.

**Figure 4:**
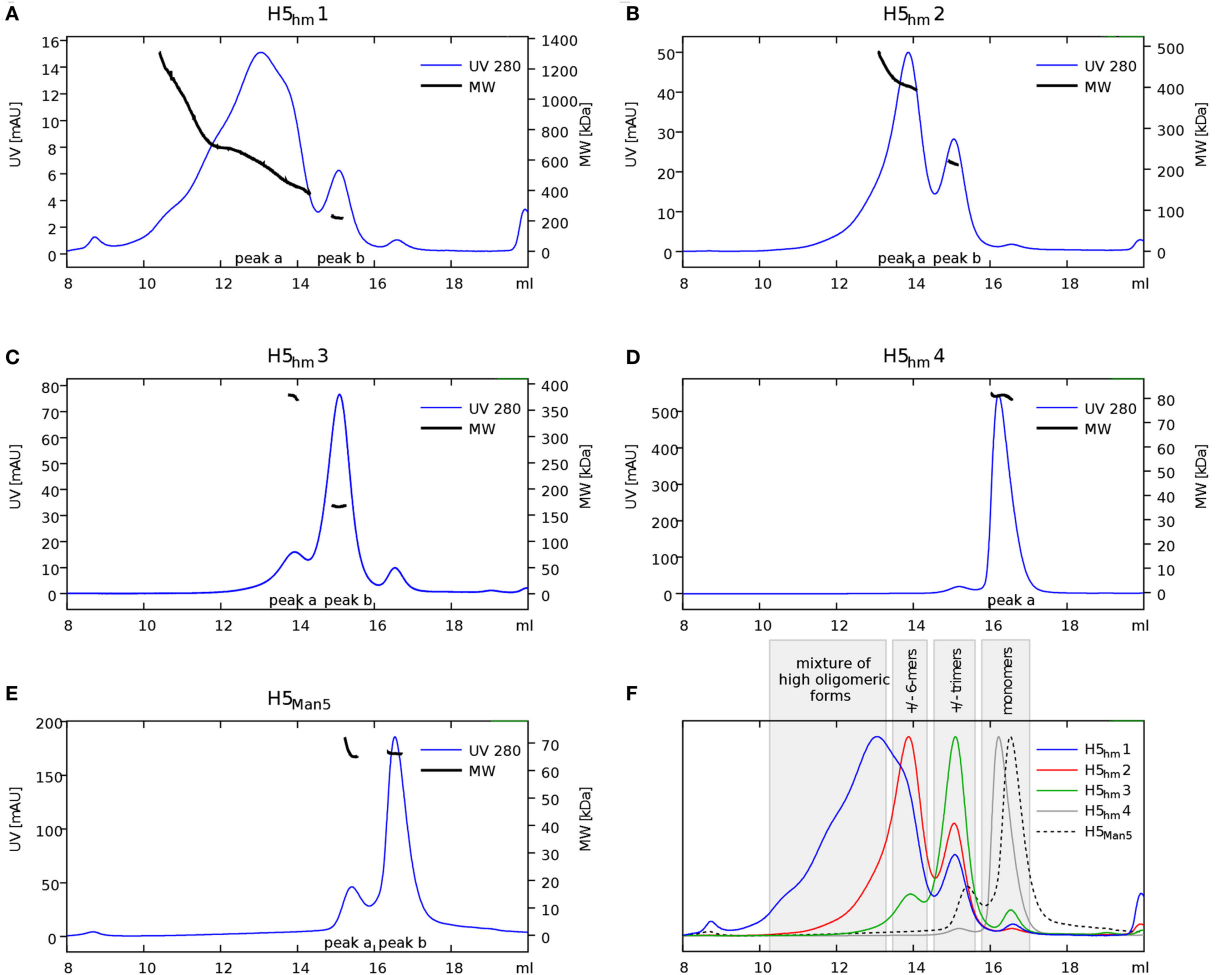
**Size exclusion chromatography (SEC)–multi-angle light scattering (MALS) analysis of H5 oligomers**. Chromatograms of H5hm **(A–D)** and H5Man5 **(E)** oligomers. Different indexes of H5hm samples (1−4) correspond to different oligomeric forms depicted in Figure 2. Molecular weights (MWs), measured by MALS, are indicated by black line. Theoretical MW value for H5 monomer is 57.8 kDa. Measured MW are higher than theoretical due to the glycosylation status of H5 antigens. All corresponding weight-averaged MWs are listed in Table 1. Overlaid chromatograms with theoretical stoichiometry predicted based on SEC–MALS results are depicted in panel **(F)**.

Size exclusion chromatography disclosed significant differences in oligomeric status of the H5_hm_ and H5_Man5_ protein variants (Figure 3). The H5_hm_ antigen was eluted in several separate peaks (Figure 3A), suggesting the presence of various oligomeric forms. Although three peaks were seen in the chromatogram of the H5_Man5_ protein (Figure 3B), SDS-PAGE analysis confirmed that the HA protein was eluted only in a single peak corresponding to the molecular mass of a monomer (Figure 3D, line B_2_). The eluted fractions were subjected to gel electrophoresis under native conditions. Native PAGE analysis revealed high MW forms in the sample of H5_hm_ (Figure 3C). Further, both H5_hm_ and H5_Man5_ monomer fractions from SEC referred to the reference band mass between 240 and 480 kDa which was much closer to the molecular mass of trimeric or tetrameric form. To better understand oligomerization status of the H5_hm_ and H5_Man5_ antigens and to gain a more precise measurements of their various oligomeric forms, we performed size exclusion chromatography coupled to multi-angle light scattering (SEC–MALS) experiments and by matrix-assisted laser desorption/ionization mass spectrometry (MALDI–MS). SEC–MALS analysis revealed that the major peak in the H5_hm_ SEC profile (LMW fraction; peak #4 in Figure 3A) contained mostly monomeric form of the H5_hm_ antigen (weight-averaged MW of 81 kDa, Figure 4D, Table 2). By contrast, HMW fractions (peaks 1−3, Figure 3A) contained higher oligomeric forms of H5_hm_. These forms ranging from dimers to trimers (peak b, Figure 4B and peak b, Figure 4C, weight-averaged MW of 167 and 215 kDa, respectively, Table 2) to pentamers/hexamers and even higher oligomers (peak a, Figure 4A, calculated MWs ranging from ~400 to ~700 kDa). This suggests that the HMW fraction on H5_hm_ antigen comprises a mixture of different oligomeric forms of the H5_hm_ protein. In contrast, the H5_Man5_ antigen contained exclusively monomeric form of the H5 protein (weight-averaged MW of 67 and 66 kDa for peaks a and b respectively, (Figure 4E) which suggests different structural conformation of the population present in peak 2, Figure 3B, Table 2). All the chromatograms with theoretical stoichiometry predicted based on SEC-MALS results were overlaid and are presented in (Figure 4F).

**Table 2:** **Weight-averaged molecular weights (MWs) of H5 oligomers calculated by size exclusion chromatography–multi-angle light scattering analysis**.

Sample	Peak	MW (kDa)	Oligomeric state
H5_hm_1	a	645	Mixture of high oligomeric forms (9-mer on average)
	b	222	Trimer
H5_hm_2	a	418	Mixture of high oligomeric forms (hexamer on average)
	b	215	Trimer
H5_hm_3	a	376	Mixture of high oligomeric forms (pentamer on average)
	b	167	Dimer
H5_hm_4	a	81	Monomer
H5_Man5_	a	67	Monomer
	b	66	Monomer

*Corresponding chromatograms are present in Figure 4*.

Matrix-assisted laser desorption/ionization mass spectrometry analysis confirmed that the LMW fraction of the H5_hm_ antigen from SEC column contains aside from monomer also dimer and trimer. In the sample of the H5_Man5_ antigen, MALDI spectrometer detected only monomers (Supplementary Material).

**Figure 5:**
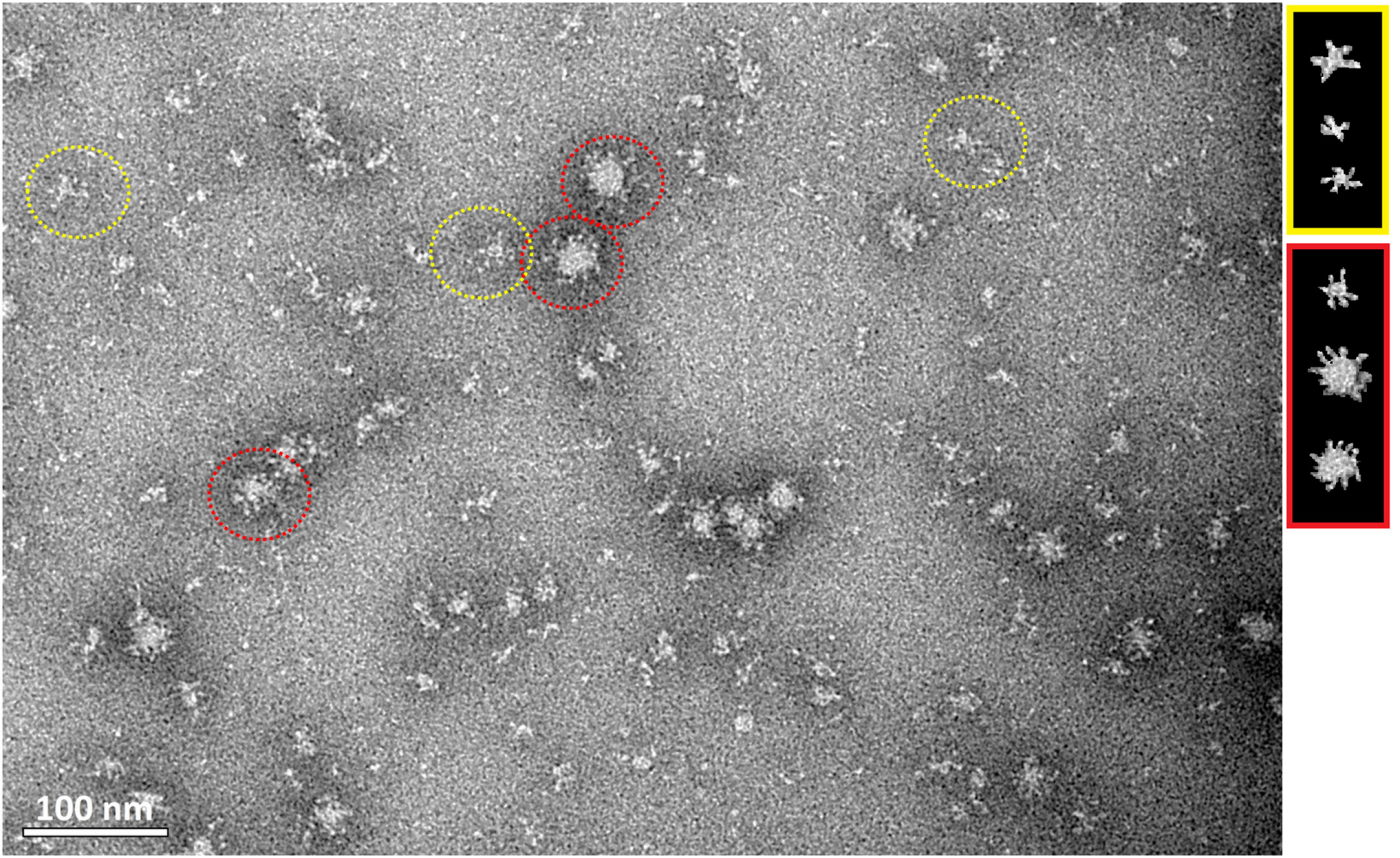
**Transmission electron microscopy of the purified H5_hm_ antigen**. Images were obtained at nominal 30,000 magnification. The white scale bar represents 100 nm. Spherical (red circle) and rosette-like structures (yellow circle) were visualized.

Transmission electron microscopy showed that HMW oligomers formed regular, spherical nanostructures with an average size of 30 nm in diameter (Figure 5). However, the morphology of the H5_hm_ nanostructures visualized by TEM is variable, e.g., rosette-like structures were also seen.

**Figure 6:**
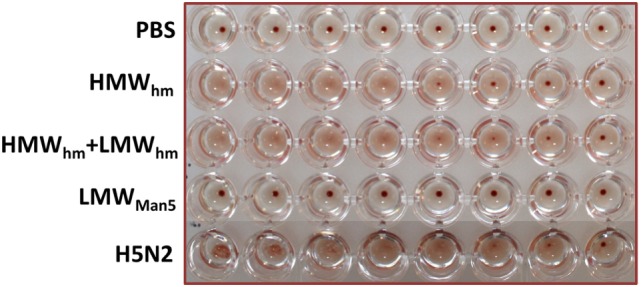
**Hemagglutination of chicken red blood cells by the H5_hm_ and H5_Man5_ proteins**. Twofold dilution of the H5 antigens were incubated with 1% chicken erythrocytes (30 min, RT). Two forms (HMW and LMW oligomers) of H5_hm_ antigen were tested. As a positive control, an inactivated H5N2 virus (A/chicken/Belgium/150/1999) was used.

The activity of the purified H5_hm_ and H5_Man5_ antigens was assessed by hemagglutination assay using chicken red blood cells. This method is a surrogate assay to measure the functionality of the influenza antigen. As a positive control, we used the inactivated A/chicken/Belgium/150/1999 H5N2 virus. Efficient HA-mediated hemagglutination was observed for the H5_hm_ HMW oligomers or a mixture of the H5_hm_ HMW and LMW oligomers. Hemagglutination test was negative for the H5_Man5_ antigen (Figure 6). This is consistent with previous data suggesting that only the high MW HA oligomers are able to bound to multiple red blood cells to create the lettices structures measured in the hemagglutination assay. The immunogenicity of the H5_hm_ and H5_Man5_ antigens were tested *in vivo*.

### *N*-Glycan-Dependent Induction of Humoral Response in Mice

**Figure 7:**
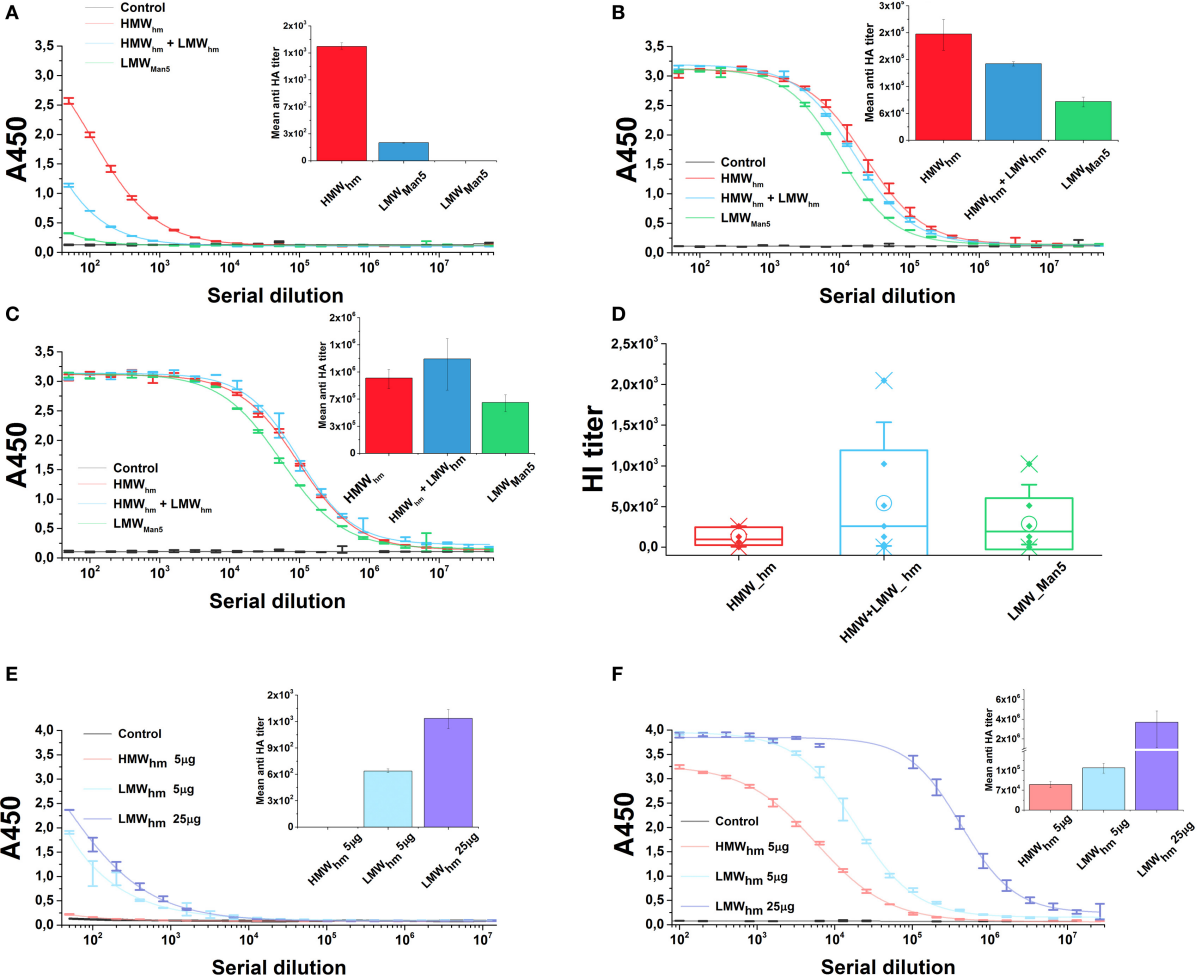
**Humoral response in mice after immunization with the H5_hm_ and H5_Man5_ proteins**. Sera samples of mice immunized with 25 µg of H5_hm_ or H5_Man5_ were pooled and the immune responses were measured in duplicate 2 weeks after immunization **(A)**, booster **(B)**, and second booster **(C)** by indirect enzyme-linked immunoabsorbant assay (ELISA) (non-linear fitting plots—bars represent SD, column plots—bars represent 95% confidence level) and 2 weeks after second booster by hemagglutination inhibition (HI) test **(D)**. Line plots represent fits for each group HI test was performed with homologous H5N1 virus (A/turkey/Poland/35/2007 H5N1, clade 2.2.3). Data for individuals (Raw data, ♦), mean (○), and the medians (—) are shown for each group. During the second immunization experiment, two doses of LMW H5_hm_ were tested. Sera samples of mice immunized twice with 5 µg of HMW H5_hm_, LMW H5_hm_, or 25 µg of H5_hm_ were measured 2 weeks after first antigen injection **(E)** and booster **(F)** by indirect ELISA (bars represent 95% confidence level).

To test the effect of the *N*-glycan length on the antigen immunogenicity, mice were immunized with both H5_hm_ and H5_Man5_ antigen variants at the same dose. Furthermore, for the H5_hm_ antigen, we tested the HMW oligomers and a mixture of HMW and LMW oligomers. Both H5_hm_ and H5_Man5_ variants elicited specific anti-HA-IgG antibodies after first administration of the vaccine; however, significant differences between these two antigens at each of measurement point were observed (Figures 7A–C). Also, the significant differences in the immunological properties between these two antigens were disclosed in HI test (Figure 7D). Although all groups were HI positive, both assays indicated that high-mannose glycosylated H5 antigen is superior to the H5_Man5_. Furthermore, what was surprising for us, both ELISA and HI assays showed that the H5_hm_ HMW oligomers mixed with LMW oligomers induced much stronger humoral response than the H5_hm_ HMW oligomers solely. The HI titers as high as 2,048 were observed in the group immunized with the mixed H5_hm_ HMW and LMW oligomers (Figure 7D). These results prompted us to follow up the immunization experiment. During the next mice immunization, we tested the H5_hm_ HMW and LMW oligomers separately and the lower dose of vaccine was applied. Mice were immunized twice with different H5_hm_ forms. Figures 7E,F present the results obtained from the ELISA test. The second immunization trial showed that one dose of LMW oligomers of the H5_hm_ antigen induced much stronger immunological response than the HMW oligomers. Further, booster injection confirmed that the LMW oligomers were more immunogenic for mice than the H5_hm_ HMW oligomers.

## Discussion

Various studies have shown that additional glycosylation sites introduced to HA molecules by site-directed mutagenesis abolish virus virulence and impact its immunogenicity (25, 26). Therefore, we decided to study the effect of *N*-glycosylation pattern without changing the number or the position of glycosylation sites. We also abandoned the idea of the addition of any foreign sequence either to enhance the oligomerization or facilitate the purification process. Although these fusion carriers are usually short peptide sequences, the changes that adding them may introduce to a protein are unpredictable. We obtained KM 71 and GlycoSwitch *Pichia* strains with stable expression of two variants of H5 antigen, with high-mannose glycosylation (H5_hm_) and low-mannose glycosylation (H5_Man5_). To test the immunological response in mice, we used three H5N1 isolates from clade 2.2. The amino acid homology between these isolates varies between 99.22 and 99.61%. The rationale for usage of three HA antigens is to mimic more natural conditions, where the circulation of genetically identical viruses is short lived, considering the fast evolution of influenza viruses. Therefore, we created a “near-homologous” setting because the H5N1 isolates used for the preparation of HA antigen, for ELISA and HI test, all belong to clade 2.2 H5N1, the predominant genotype during 2005/2006 epidemic of HPAI. Our immunization experiments showed that the H5_hm_ antigen induced significantly stronger HA-antibody response than the H5_Man5_ antigen. The mean anti-HA antibodies titer as high as 1.5 × 10^6^ after the third dose of the H5_hm_ vaccine was detected. However, it is well known that protective immunity correlates with HI titers rather than antigen-binding antibodies. Indeed, HI tests showed that the antigen with high-mannose glycosylation gave higher neutralizing antibodies titer than the low-glycosylated H5 antigen. It was reported by Lin and colleagues that recombinant H5 antigen with high-mannose glycans was highly immunogenic and elicited strong immunological response (27). Recently, Liu and colleagues showed a very positive effect of high-mannose type glycosylation on the immunogenicity of recombinant H1, H5, and H7 antigens (28). On the other hand, Chen and colleagues reported that monoglycosylated H1 induced higher HI-antibodies titer and exhibited higher neutralizing capacity than fully glycosylated HA (29). The same effect was observed for the H5 protein (12). However, in above studies, antigen variants were forced to form trimers since the transmembrane domain was replaced with the residues that are trimerization segment.

In our study, the differences in the immunological properties may be explained by the differences in the oligomeric status of the H5_hm_ and H5_Man5_ antigens. Structural analysis showed that the high-mannose glycosylated antigen but not Man_5_ oligomerized into spherical like structures. The H5_hm_ antigen also formed low molecular oligomers like dimers/trimers and these oligomers combined with monomers were more immunogenic for mice than high MW oligomers. For the H5_Man5_ antigen, only monomeric forms were detected. Gallagher and colleagues demonstrated that glycosylation state had an impact on hemagglutinin oligomerization. They showed that although no individual oligosaccharide side chain was necessary or sufficient for the folding, the mutant HAs having less than five oligosaccharides formed intracellular aggregates (18). The differences in oligomeric status of the antigens may be also caused by the number of glycosylated sites in the antigens. Mass spectrometry analysis confirmed that four sites in H5_hm_ and three sites in H5_Man5_ are glycosylated. Without any mutation, N165 is not glycosylated in the H5_Man5_ antigen. Further studies are necessary to explore the effect of the *N*-glycosylation on the recombinant hemagglutinin oligomerization.

A low-cost production of vaccine with the specific match to the genetics of current outbreak’s virus is strongly required. In this study, we used the *P. pastoris* cells to produce a soluble H5 antigens. The *P. pastoris* expression system has been commonly utilized as a platform to produce various proteins significant for medical industry, including vaccine antigens (Shanvac™, Elovac™, Gavac™). The another crucial issue for an influenza vaccine to be licensed for use is a development of efficient process for the purification of the protein product. We optimized both of vaccine production steps (expression and purification); however, the efficient production process was achieved only for high-mannose H5 protein. The efficiency of the H5_Man5_ antigen production was rather low, thus the utilization of GlycoSwitch^®^ *P. pastoris* strain as a vaccine platform is unattractive. For the high-mannose H5 antigen, which was proved to be much more immunogenic, the excellent efficiency up to 150 mg of highly purified protein from 1 l of culture medium (6,000 doses) was obtained. This efficiency presumably could be easily scaled up using bioreactors.

In summary, the *N*-glycosylation influences the biological properties of the influenza H5 antigens. The presence and the number of carbohydrate moieties (mannose) had an impact on the oligomerization and the immunogenicity of the H5_hm_ and H5_Man5_ antigens. Structural analysis showed that the high-mannose glycosylated antigen not only formed low molecular oligomers like dimers and trimers but also oligomerized into spherical structures similar to influenza virions. The high-mannose H5 antigen induced significantly stronger HA-antibody response than the H5_Man5_ antigen. These results might be highly relevant for the influenza vaccine design.

## Ethics Statement

Immunization studies were conducted at the Institute of Experimental Medicine PAS (Warsaw, Poland) under the control of Bioethics Committee (Permission No 21/2014).

## Author Contributions

AM and MP performed the experiments, MC-C conducted SEC–MALS analysis, PK conducted mice experiment. ZM and KŚ contributed H5N1 virus and performed HI analysis. AM, MP, and EK participated in study design and data analysis. All authors participated in manuscript and figures preparation and have read and approved the final manuscript.

## Conflict of Interest Statement

The authors declare that the research was conducted in the absence of any commercial or financial relationships that could be construed as a potential conflict of interest.

## Abbreviations

HA, hemagglutinin; Man, mannose; GlcNA, *N*-acetylglucoseamine; HexNAc, *N*-acetylhexoseamine; AOX, alcohol oxidase; Endo H, endoglycosidase H; PAGE, polyacrylamide gel electrophoresis; IEC, ion exchange chromatography; SEC, size exclusion chromatography; MALS, multi-angle light scattering; MALDI–MS, matrix-assisted laser desorption/ionization mass spectrometry; TEM, transmission electron microscopy; HI, hemagglutination inhibition.
