# Supplementary material for: The Length of N-Glycans of Recombinant H5N1 Hemagglutinin Influences the Oligomerization and Immunogenicity of Vaccine Antigen
Source: Front Immunol. 2017 Apr 20;8:444. doi: 10.3389/fimmu.2017.00444 (PMC5397403; doi:10.3389/fimmu.2017.00444)
Supplement: Supplementary file 1 [file Data_Sheet_1.DOCX]

Supplementary Material

The length of *N*-glycans of recombinant H5N1 hemagglutinin influences the oligomerization and immunogenicity
of vaccine antigen

Agnieszka Macioła, Maria Pietrzak, Piotr Kosson, Mariusz Czarnocki-Cieciura, Krzysztof Śmietanka, Zenon Minta, Edyta Kopera*

*** Correspondence:** Edyta Kopera: ekopera@ibb.waw.pl

# Supplementary

# Acquired MS/MS raw data were processed by Mascot Distiller followed by Mascot Search (Matrix Science, London, UK, on-site license) against users defined database. Search parameters for precursor and product ions mass tolerance were 20 ppm and 0.1 Da, respectively, enzyme specificity: trypsin, missed cleavage sites allowed: 1, fixed modification of cysteine by carbamidomethylation and variable modification of methionine oxidation and HexNAc at asparagine. Peptides with Mascot Score exceeding the threshold value corresponding to < 1% False Positive Rate, calculated by Mascot procedure were considered to be positively identified.

# Mascot Search Results. Peptide report for H5_hm_

| Query | Observed | Mr(expt) | Mr(calc) | ppm | Miss | Score | Expect | Rank | Unique | Peptide |
| --- | --- | --- | --- | --- | --- | --- | --- | --- | --- | --- |
| 2696 | 835.9362 | 1669.8579 | 1669.8523 | 3.35 | 0 | 66 | 2.8e-07 | 1 | U | K.NVTVTHAQDILEK.T + HexNAc (N) |
| 3058 | 948.4013 | 1894.7881 | 1894.7857 | 1.26 | 0 | 87 | 1.9e-09 | 1 | U | R.NGTYDYPQYSEEAR.L + HexNAc (N) |
| 4218 | 1074.8563 | 3221.5470 | 3221.5308 | 5.03 | 0 | 72 | 6e-08 | 1 | U | K.CQTPIGAINSSMPFHNIHPLTIGECPK.Y + Oxidation (M); HexNAc (N) |
| 4329 | 1113.5386 | 3337.5938 | 3337.5938 | 10.2 | 0 | 79 | 1.3e-08 | 1 | U | R.SYNNTNQEDLLVLWGIHHPNDAAEQTR.L + HexNAc (N) |

## Supplementary Figures


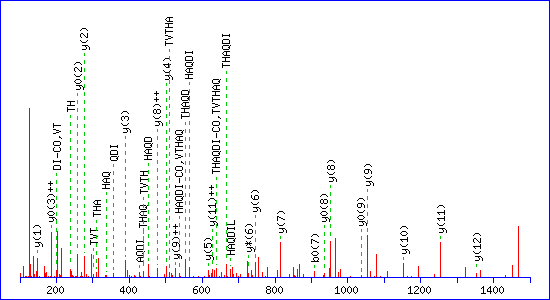


**Figure S1.** **MS/MS Fragmentation of NVTVTHAQDILEK.**

Monoisotopic mass of neutral peptide Mr(calc): 1669.8523. Ions score: 66

**Table S1.** Theoretical fragmentation of a peptide NVTVTHAQDILEK. Ions identified in the experimental MS/MS spectrum by the Mascot software are marked red

| **#** | **a** | **a^++^** | **b** | **b^++^** | **b*** | **b*^++^** | **b^0^** | **b^0++^** | **Seq.** | **y** | **y^++^** | **y*** | **y*^++^** | **y^0^** | **y^0++^** | **#** |
| --- | --- | --- | --- | --- | --- | --- | --- | --- | --- | --- | --- | --- | --- | --- | --- | --- |
| **1** | 290.1347 | 145.5710 | 318.1296 | 159.5684 | 301.1030 | 151.0552 |  |  | **N** |  |  |  |  |  |  | **13** |
| **2** | 389.2031 | 195.1052 | 417.1980 | 209.1026 | 400.1714 | 200.5894 |  |  | **V** | ***1353.7373*** | 677.3723 | 1336.7107 | 668.8590 | 1335.7267 | 668.3670 | **12** |
| **3** | 490.2508 | 245.6290 | 518.2457 | 259.6265 | 501.2191 | 251.1132 | 500.2351 | 250.6212 | **T** | ***1254.6688*** | 627.8381 | 1237.6423 | 619.3248 | 1236.6583 | 618.8328 | **11** |
| **4** | 589.3192 | 295.1632 | 617.3141 | 309.1607 | 600.2875 | 300.6474 | 599.3035 | 300.1554 | **V** | ***1153.6212*** | 577.3142 | 1136.5946 | 568.8009 | 1135.6106 | 568.3089 | **10** |
| **5** | 690.3668 | 345.6871 | 718.3618 | 359.6845 | 701.3352 | 351.1712 | 700.3512 | 350.6792 | **T** | ***1054.5528*** | 527.7800 | 1037.5262 | 519.2667 | 1036.5422 | 518.7747 | **9** |
| **6** | 827.4258 | 414.2165 | 855.4207 | **428.2140** | 838.3941 | 419.7007 | 837.4101 | 419.2087 | **H** | ***953.5051*** | 477.2562 | 936.4785 | 468.7429 | 935.4945 | 468.2509 | **8** |
| **7** | 898.4629 | 449.7351 | 926.4578 | 463.7325 | 909.4312 | 455.2193 | 908.4472 | 454.7272 | **A** | ***816.4462*** | 408.7267 | 799.4196 | 400.2134 | 798.4356 | 399.7214 | **7** |
| **8** | 1026.5215 | 513.7644 | 1054.5164 | 527.7618 | 1037.4898 | 519.2485 | 1036.5058 | 518.7565 | **Q** | ***745.4090*** | 373.2082 | 728.3825 | 364.6949 | 727.3985 | 364.2029 | **6** |
| **9** | 1141.5484 | 571.2778 | 1169.5433 | 585.2753 | 1152.5168 | 576.7620 | 1151.5327 | 576.2700 | **D** | ***617.3505*** | 309.1789 | 600.3239 | 300.6656 | 599.3399 | 300.1736 | **5** |
| **10** | 1254.6325 | 627.8199 | 1282.6274 | 641.8173 | 1265.6008 | 633.3041 | 1264.6168 | 632.8120 | **I** | ***502.3235*** | 251.6654 | 485.2970 | 243.1521 | 484.3130 | 242.6601 | **4** |
| **11** | 1367.7165 | 684.3619 | 1395.7114 | 698.3594 | 1378.6849 | 689.8461 | 1377.7009 | 689.3541 | **L** | ***389.2395*** | 195.1234 | 372.2129 | 186.6101 | 371.2289 | 186.1181 | **3** |
| **12** | 1496.7591 | 748.8832 | 1524.7540 | 762.8807 | 1507.7275 | 754.3674 | 1506.7435 | 753.8754 | **E** | ***276.1554*** | 138.5813 | 259.1288 | 130.0681 | 258.1448 | 129.5761 | **2** |
| **13** |  |  |  |  |  |  |  |  | **K** | ***147.1128*** | 74.0600 | 130.0863 | 65.5468 |  |  | **1** |


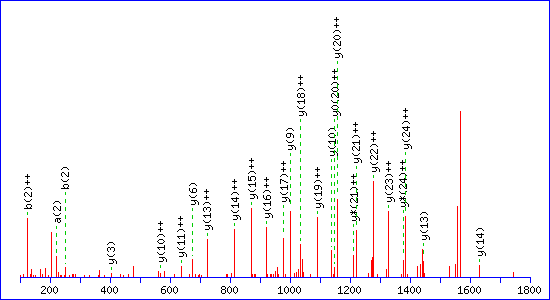


**Figure S2.** **MS/MS Fragmentation of SYNNTNQEDLLVLWGIHHPNDAAEQTR.** Monoisotopic mass of neutral peptide Mr(calc): 3337.5599. Ions score: 79

**Table S2**. Theoretical fragmentation of a peptide SYNNTNQEDLLVLWGIHHPNDAAEQTR. Ions identified in the experimental MS/MS spectrum by the Mascot software are marked red

| **#** | **a** | **a^++^** | **b** | **b^++^** | **b*** | **b*^++^** | **b^0^** | **b^0++^** | **Seq.** | **y** | **y^++^** | **y*** | **y*^++^** | **y^0^** | **y^0++^** | **#** |
| --- | --- | --- | --- | --- | --- | --- | --- | --- | --- | --- | --- | --- | --- | --- | --- | --- |
| **1** | 60.0444 | 30.5258 | 88.0393 | 44.5233 |  |  | 70.0287 | 35.5180 | **S** |  |  |  |  |  |  | **27** |
| **2** | **223.1077** | 112.0575 | **251.1026** | **126.0550** |  |  | 233.0921 | 117.0497 | **Y** | 3251.5352 | 1626.2712 | 3234.5087 | 1617.7580 | 3233.5246 | 1617.2660 | **26** |
| **3** | 540.2300 | 270.6186 | 568.2249 | 284.6161 | 551.1984 | 276.1028 | 550.2144 | 275.6108 | **N** | 3088.4719 | 1544.7396 | 3071.4453 | 1536.2263 | 3070.4613 | 1535.7343 | **25** |
| **4** | 654.2729 | 327.6401 | 682.2679 | 341.6376 | 665.2413 | 333.1243 | 664.2573 | 332.6323 | **N** | 2771.3496 | 1386.1784 | 2754.3230 | 1377.6652 | 2753.3390 | 1377.1731 | **24** |
| **5** | 755.3206 | 378.1640 | 783.3155 | 392.1614 | 766.2890 | 383.6481 | 765.3050 | 383.1561 | **T** | 2657.3067 | 1329.1570 | 2640.2801 | 1320.6437 | 2639.2961 | 1320.1517 | **23** |
| **6** | 869.3636 | 435.1854 | 897.3585 | 449.1829 | 880.3319 | 440.6696 | 879.3479 | 440.1776 | **N** | 2556.2590 | 1278.6331 | 2539.2324 | 1270.1199 | 2538.2484 | 1269.6278 | **22** |
| **7** | 997.4221 | 499.2147 | 1025.4170 | 513.2122 | 1008.3905 | 504.6989 | 1007.4065 | 504.2069 | **Q** | 2442.2160 | 1221.6117 | 2425.1895 | 1213.0984 | 2424.2055 | 1212.6064 | **21** |
| **8** | 1126.4647 | 563.7360 | 1154.4596 | 577.7335 | 1137.4331 | 569.2202 | 1136.4491 | 568.7282 | **E** | 2314.1575 | 1157.5824 | 2297.1309 | 1149.0691 | 2296.1469 | 1148.5771 | **20** |
| **9** | 1241.4917 | 621.2495 | 1269.4866 | 635.2469 | 1252.4600 | 626.7337 | 1251.4760 | 626.2416 | **D** | 2185.1149 | 1093.0611 | 2168.0883 | 1084.5478 | 2167.1043 | 1084.0558 | **19** |
| **10** | 1354.5757 | 677.7915 | 1382.5706 | 691.7890 | 1365.5441 | 683.2757 | 1364.5601 | 682.7837 | **L** | 2070.0879 | 1035.5476 | 2053.0614 | 1027.0343 | 2052.0774 | 1026.5423 | **18** |
| **11** | 1467.6598 | 734.3335 | 1495.6547 | 748.3310 | 1478.6282 | 739.8177 | 1477.6441 | 739.3257 | **L** | 1957.0039 | 979.0056 | 1939.9773 | 970.4923 | 1938.9933 | 970.0003 | **17** |
| **12** | 1566.7282 | 783.8677 | 1594.7231 | 797.8652 | 1577.6966 | 789.3519 | 1576.7126 | 788.8599 | **V** | 1843.9198 | 922.4635 | 1826.8933 | 913.9503 | 1825.9092 | 913.4583 | **16** |
| **13** | 1679.8123 | 840.4098 | 1707.8072 | 854.4072 | 1690.7806 | 845.8940 | 1689.7966 | 845.4019 | **L** | 1744.8514 | 872.9293 | 1727.8248 | 864.4161 | 1726.8408 | 863.9241 | **15** |
| **14** | 1865.8916 | 933.4494 | 1893.8865 | 947.4469 | 1876.8600 | 938.9336 | 1875.8759 | 938.4416 | **W** | **1631.7673** | 816.3873 | 1614.7408 | 807.8740 | 1613.7568 | 807.3820 | **14** |
| **15** | 1922.9130 | 961.9602 | 1950.9080 | 975.9576 | 1933.8814 | 967.4443 | 1932.8974 | 966.9523 | **G** | **1445.6880** | 723.3476 | 1428.6615 | 714.8344 | 1427.6775 | 714.3424 | **13** |
| **16** | 2035.9971 | 1018.5022 | 2063.9920 | 1032.4997 | 2046.9655 | 1023.9864 | 2045.9815 | 1023.4944 | **I** | 1388.6666 | 694.8369 | 1371.6400 | 686.3236 | 1370.6560 | 685.8316 | **12** |
| **17** | 2173.0560 | 1087.0316 | 2201.0509 | 1101.0291 | 2184.0244 | 1092.5158 | 2183.0404 | 1092.0238 | **H** | 1275.5825 | 638.2949 | 1258.5559 | 629.7816 | 1257.5719 | 629.2896 | **11** |
| **18** | 2310.1149 | 1155.5611 | 2338.1099 | 1169.5586 | 2321.0833 | 1161.0453 | 2320.0993 | 1160.5533 | **H** | **1138.5236** | 569.7654 | 1121.4970 | 561.2522 | 1120.5130 | 560.7601 | **10** |
| **19** | 2407.1677 | 1204.0875 | 2435.1626 | 1218.0849 | 2418.1361 | 1209.5717 | 2417.1520 | 1209.0797 | **P** | **1001.4647** | 501.2360 | 984.4381 | 492.7227 | 983.4541 | 492.2307 | **9** |
| **20** | 2521.2106 | 1261.1090 | 2549.2055 | 1275.1064 | 2532.1790 | 1266.5931 | 2531.1950 | 1266.1011 | **N** | 904.4119 | 452.7096 | 887.3854 | 444.1963 | 886.4013 | 443.7043 | **8** |
| **21** | 2636.2376 | 1318.6224 | 2664.2325 | 1332.6199 | 2647.2059 | 1324.1066 | 2646.2219 | 1323.6146 | **D** | 790.3690 | 395.6881 | 773.3424 | 387.1749 | 772.3584 | 386.6828 | **7** |
| **22** | 2707.2747 | 1354.1410 | 2735.2696 | 1368.1384 | 2718.2430 | 1359.6252 | 2717.2590 | 1359.1332 | **A** | **675.3420** | 338.1747 | 658.3155 | 329.6614 | 657.3315 | 329.1694 | **6** |
| **23** | 2778.3118 | 1389.6595 | 2806.3067 | 1403.6570 | 2789.2802 | 1395.1437 | 2788.2961 | 1394.6517 | **A** | 604.3049 | 302.6561 | 587.2784 | 294.1428 | 586.2944 | 293.6508 | **5** |
| **24** | 2907.3544 | 1454.1808 | 2935.3493 | 1468.1783 | 2918.3228 | 1459.6650 | 2917.3387 | 1459.1730 | **E** | 533.2678 | 267.1375 | 516.2413 | 258.6243 | 515.2572 | 258.1323 | **4** |
| **25** | 3035.4130 | 1518.2101 | 3063.4079 | 1532.2076 | 3046.3813 | 1523.6943 | 3045.3973 | 1523.2023 | **Q** | **404.2252** | 202.6162 | 387.1987 | 194.1030 | 386.2146 | 193.6110 | **3** |
| **26** | 3136.4606 | 1568.7340 | 3164.4556 | 1582.7314 | 3147.4290 | 1574.2181 | 3146.4450 | 1573.7261 | **T** | 276.1666 | 138.5870 | 259.1401 | 130.0737 | 258.1561 | 129.5817 | **2** |
| **27** |  |  |  |  |  |  |  |  | **R** | 175.1190 | 88.0631 | 158.0924 | 79.5498 |  |  | **1** |


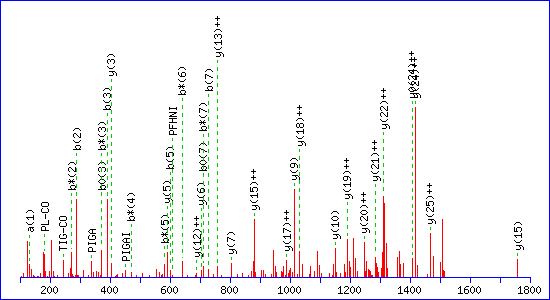


**Figure S3.** **MS/MS Fragmentation of CQTPIGAINSSMPFHNIHPLTIGECPK.**

Monoisotopic mass of neutral peptide Mr(calc): 3221.53. Ions score: 72

**Table S3.** Theoretical fragmentation of a peptide CQTPIGAINSSMPFHNIHPLTIGECPK. Ions identified in the experimental MS/MS spectrum by the Mascot software are marked red.

| **#** | **a** | **a^++^** | **b** | **b^++^** | **b*** | **b*^++^** | **b^0^** | **b^0++^** | **Seq.** | **y** | **y^++^** | **y*** | **y*^++^** | **y^0^** | **y^0++^** | **#** |
| --- | --- | --- | --- | --- | --- | --- | --- | --- | --- | --- | --- | --- | --- | --- | --- | --- |
| **1** | **133.0430** | 67.0251 | 161.0379 | 81.0226 |  |  |  |  | **C** |  |  |  |  |  |  | **27** |
| **2** | 261.1016 | 131.0544 | **289.0965** | 145.0519 | 272.0700 | 136.5386 |  |  | **Q** | 3062.5074 | 1531.7573 | 3045.4809 | 1523.2441 | 3044.4968 | 1522.7521 | **26** |
| **3** | 362.1493 | 181.5783 | **390.1442** | 195.5757 | 373.1176 | 187.0625 | 372.1336 | 186.5704 | **T** | 2934.4488 | 1467.7281 | 2917.4223 | 1459.2148 | 2916.4383 | 1458.7228 | **25** |
| **4** | 459.2020 | 230.1047 | 487.1969 | 244.1021 | 470.1704 | 235.5888 | 469.1864 | 235.0968 | **P** | 2833.4011 | 1417.2042 | 2816.3746 | 1408.6909 | 2815.3906 | 1408.1989 | **24** |
| **5** | 572.2861 | 286.6467 | **600.2810** | 300.6441 | 583.2545 | 292.1309 | 582.2704 | 291.6389 | **I** | 2736.3484 | 1368.6778 | 2719.3218 | 1360.1646 | 2718.3378 | 1359.6725 | **23** |
| **6** | 629.3076 | 315.1574 | 657.3025 | 329.1549 | 640.2759 | 320.6416 | 639.2919 | 320.1496 | **G** | 2623.2643 | 1312.1358 | 2606.2378 | 1303.6225 | 2605.2538 | 1303.1305 | **22** |
| **7** | 700.3447 | 350.6760 | **728.3396** | 364.6734 | 711.3130 | 356.1602 | 710.3290 | 355.6681 | **A** | 2566.2429 | 1283.6251 | 2549.2163 | 1275.1118 | 2548.2323 | 1274.6198 | **21** |
| **8** | 813.4287 | 407.2180 | 841.4237 | 421.2155 | 824.3971 | 412.7022 | 823.4131 | 412.2102 | **I** | 2495.2057 | 1248.1065 | 2478.1792 | 1239.5932 | 2477.1952 | 1239.1012 | **20** |
| **9** | 1130.5510 | 565.7792 | 1158.5460 | 579.7766 | 1141.5194 | 571.2633 | 1140.5354 | 570.7713 | **N** | 2382.1217 | 1191.5645 | 2365.0951 | 1183.0512 | 2364.1111 | 1182.5592 | **19** |
| **10** | 1217.5831 | 609.2952 | 1245.5780 | 623.2926 | 1228.5514 | 614.7794 | 1227.5674 | 614.2873 | **S** | 2064.9994 | 1033.0033 | 2047.9728 | 1024.4901 | 2046.9888 | 1023.9980 | **18** |
| **11** | 1304.6151 | 652.8112 | 1332.6100 | 666.8086 | 1315.5835 | 658.2954 | 1314.5994 | 657.8034 | **S** | 1977.9674 | 989.4873 | 1960.9408 | 980.9740 | 1959.9568 | 980.4820 | **17** |
| **12** | 1435.6556 | 718.3314 | 1463.6505 | 732.3289 | 1446.6239 | 723.8156 | 1445.6399 | 723.3236 | **M** | 1890.9353 | 945.9713 | 1873.9088 | 937.4580 | 1872.9248 | 936.9660 | **16** |
| **13** | 1532.7083 | 766.8578 | 1560.7033 | 780.8553 | 1543.6767 | 772.3420 | 1542.6927 | 771.8500 | **P** | ***1759.8948*** | 880.4511 | 1742.8683 | 871.9378 | 1741.8843 | 871.4458 | **15** |
| **14** | 1679.7768 | 840.3920 | 1707.7717 | 854.3895 | 1690.7451 | 845.8762 | 1689.7611 | 845.3842 | **F** | 1662.8421 | 831.9247 | 1645.8155 | 823.4114 | 1644.8315 | 822.9194 | **14** |
| **15** | 1816.8357 | 908.9215 | 1844.8306 | 922.9189 | 1827.8040 | 914.4057 | 1826.8200 | 913.9136 | **H** | 1515.7737 | 758.3905 | 1498.7471 | 749.8772 | 1497.7631 | 749.3852 | **13** |
| **16** | 1930.8786 | 965.9429 | 1958.8735 | 979.9404 | 1941.8470 | 971.4271 | 1940.8629 | 970.9351 | **N** | 1378.7147 | 689.8610 | 1361.6882 | 681.3477 | 1360.7042 | 680.8557 | **12** |
| **17** | 2043.9627 | 1022.4850 | 2071.9576 | 1036.4824 | 2054.9310 | 1027.9692 | 2053.9470 | 1027.4771 | **I** | 1264.6718 | 632.8395 | 1247.6453 | 624.3263 | 1246.6613 | 623.8343 | **11** |
| **18** | 2181.0216 | 1091.0144 | 2209.0165 | 1105.0119 | 2191.9899 | 1096.4986 | 2191.0059 | 1096.0066 | **H** | ***1151.5878*** | 576.2975 | 1134.5612 | 567.7842 | 1133.5772 | 567.2922 | **10** |
| **19** | 2278.0743 | 1139.5408 | 2306.0693 | 1153.5383 | 2289.0427 | 1145.0250 | 2288.0587 | 1144.5330 | **P** | ***1014.5288*** | 507.7681 | 997.5023 | 499.2548 | 996.5183 | 498.7628 | **9** |
| **20** | 2391.1584 | 1196.0828 | 2419.1533 | 1210.0803 | 2402.1268 | 1201.5670 | 2401.1427 | 1201.0750 | **L** | 917.4761 | 459.2417 | 900.4495 | 450.7284 | 899.4655 | 450.2364 | **8** |
| **21** | 2492.2061 | 1246.6067 | 2520.2010 | 1260.6041 | 2503.1744 | 1252.0909 | 2502.1904 | 1251.5989 | **T** | ***804.3920*** | 402.6996 | 787.3655 | 394.1864 | 786.3815 | 393.6944 | **7** |
| **22** | 2605.2901 | 1303.1487 | 2633.2851 | 1317.1462 | 2616.2585 | 1308.6329 | 2615.2745 | 1308.1409 | **I** | ***703.3443*** | 352.1758 | 686.3178 | 343.6625 | 685.3338 | 343.1705 | **6** |
| **23** | 2662.3116 | 1331.6594 | 2690.3065 | 1345.6569 | 2673.2800 | 1337.1436 | 2672.2960 | 1336.6516 | **G** | ***590.2603*** | 295.6338 | 573.2337 | 287.1205 | 572.2497 | 286.6285 | **5** |
| **24** | 2791.3542 | 1396.1807 | 2819.3491 | 1410.1782 | 2802.3226 | 1401.6649 | 2801.3385 | 1401.1729 | **E** | 533.2388 | 267.1230 | 516.2123 | 258.6098 | 515.2282 | 258.1178 | **4** |
| **25** | 2951.3848 | 1476.1961 | 2979.3798 | 1490.1935 | 2962.3532 | 1481.6802 | 2961.3692 | 1481.1882 | **C** | ***404.1962*** | 202.6017 | 387.1697 | 194.0885 |  |  | **3** |
| **26** | 3048.4376 | 1524.7224 | 3076.4325 | 1538.7199 | 3059.4060 | 1530.2066 | 3058.4220 | 1529.7146 | **P** | ***244.1656*** | 122.5864 | 227.1390 | 114.0731 |  |  | **2** |
| **27** |  |  |  |  |  |  |  |  | **K** | 147.1128 | 74.0600 | 130.0863 | 65.5468 |  |  | **1** |


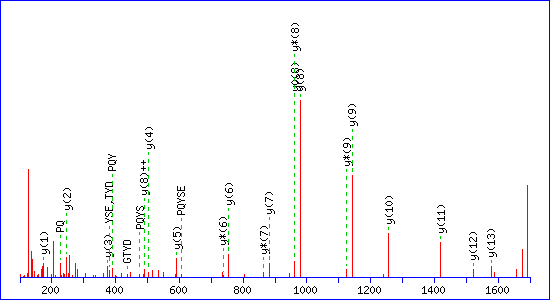


**Figure S4.** **MS/MS Fragmentation of NGTYDYPQYSEEAR.**

Monoisotopic mass of neutral peptide Mr(calc): 1894.78. Ions score: 87

**Table S4.** Theoretical fragmentation of a peptide NGTYDYPQYSEEAR. Ions identified in the experimental MS/MS spectrum by the Mascot software are marked red.

| **#** | **a** | **a^++^** | **b** | **b^++^** | **b*** | **b*^++^** | **b^0^** | **b^0++^** | **Seq.** | **y** | **y^++^** | **y*** | **y*^++^** | **y^0^** | **y^0++^** | **#** |
| --- | --- | --- | --- | --- | --- | --- | --- | --- | --- | --- | --- | --- | --- | --- | --- | --- |
| **1** | 290.1347 | 145.5710 | 318.1296 | 159.5684 | 301.1030 | 151.0552 |  |  | **N** |  |  |  |  |  |  | **14** |
| **2** | 347.1561 | 174.0817 | 375.1510 | 188.0792 | 358.1245 | 179.5659 |  |  | **G** | ***1578.6707*** | 789.8390 | 1561.6441 | 781.3257 | 1560.6601 | 780.8337 | **13** |
| **3** | 448.2038 | 224.6055 | 476.1987 | 238.6030 | 459.1722 | 230.0897 | 458.1882 | 229.5977 | **T** | ***1521.6492*** | 761.3283 | 1504.6227 | 752.8150 | 1503.6387 | 752.3230 | **12** |
| **4** | 611.2671 | 306.1372 | 639.2620 | 320.1347 | 622.2355 | 311.6214 | 621.2515 | 311.1294 | **Y** | ***1420.6016*** | 710.8044 | 1403.5750 | 702.2911 | 1402.5910 | 701.7991 | **11** |
| **5** | 726.2941 | 363.6507 | 754.2890 | 377.6481 | 737.2624 | 369.1349 | 736.2784 | 368.6429 | **D** | ***1257.5382*** | 629.2727 | 1240.5117 | 620.7595 | 1239.5277 | 620.2675 | **10** |
| **6** | 889.3574 | 445.1823 | 917.3523 | 459.1798 | 900.3258 | 450.6665 | 899.3418 | 450.1745 | **Y** | ***1142.5113*** | 571.7593 | 1125.4847 | 563.2460 | 1124.5007 | 562.7540 | **9** |
| **7** | 986.4102 | 493.7087 | 1014.4051 | 507.7062 | 997.3785 | 499.1929 | 996.3945 | 498.7009 | **P** | ***979.4480*** | 490.2276 | 962.4214 | 481.7143 | 961.4374 | 481.2223 | **8** |
| **8** | 1114.4687 | 557.7380 | 1142.4637 | 571.7355 | 1125.4371 | 563.2222 | 1124.4531 | 562.7302 | **Q** | ***882.3952*** | 441.7012 | 865.3686 | 433.1880 | 864.3846 | 432.6959 | **7** |
| **9** | 1277.5321 | 639.2697 | 1305.5270 | 653.2671 | 1288.5004 | 644.7539 | 1287.5164 | 644.2619 | **Y** | ***754.3366*** | 377.6719 | 737.3101 | 369.1587 | 736.3260 | 368.6667 | **6** |
| **10** | 1364.5641 | 682.7857 | 1392.5590 | 696.7831 | 1375.5325 | 688.2699 | 1374.5485 | 687.7779 | **S** | ***591.2733*** | 296.1403 | 574.2467 | 287.6270 | 573.2627 | 287.1350 | **5** |
| **11** | 1493.6067 | 747.3070 | 1521.6016 | 761.3044 | 1504.5751 | 752.7912 | 1503.5910 | 752.2992 | **E** | ***504.2413*** | 252.6243 | 487.2147 | 244.1110 | 486.2307 | 243.6190 | **4** |
| **12** | 1622.6493 | 811.8283 | 1650.6442 | 825.8257 | 1633.6177 | 817.3125 | 1632.6336 | 816.8205 | **E** | ***375.1987*** | 188.1030 | 358.1721 | 179.5897 | 357.1881 | 179.0977 | **3** |
| **13** | 1693.6864 | 847.3468 | 1721.6813 | 861.3443 | 1704.6548 | 852.8310 | 1703.6708 | 852.3390 | **A** | ***246.1561*** | 123.5817 | 229.1295 | 115.0684 |  |  | **2** |
| **14** |  |  |  |  |  |  |  |  | **R** | ***175.1190*** | 88.0631 | 158.0924 | 79.5498 |  |  | **1** |

**Mascot Search Results. Peptide report for H5_Man5_**

| Query | Observed | Mr(expt) | Mr(calc) | ppm | Miss | Score | Expect | Rank | Unique | Peptide |
| --- | --- | --- | --- | --- | --- | --- | --- | --- | --- | --- |
| 4104 | 835.9343 | 1669.8539 | 1669.8523 | 1.00 | 0 | 85 | 3.4e-09 | 1 | U | K.NVTVTHAQDILEK.T + HexNAc (N) |
| 4586 | 948.3995 | 1894.7844 | 1894.7857 | -0.68 | 0 | 80 | 9.2e-09 | 1 | U | R.NGTYDYPQYSEEAR.L + HexNAc (N) |
| 5809 | 810.3895 | 3237.5287 | 3237.5257 | 0.94 | 0 | 65 | 3.1e-07 | 1 | U | K.CQTPIGAINSSMPFHNIHPLTIGECPK.Y + Oxidation (M); HexNAc (N) |


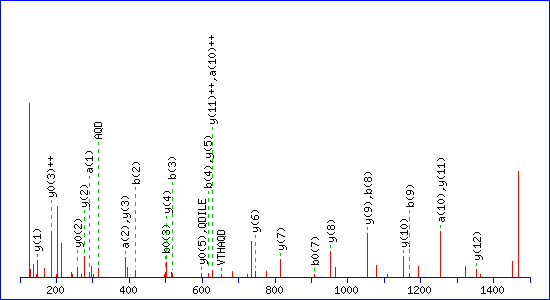


**Figure S5.** **MS/MS Fragmentation of NVTVTHAQDILEK.**
Monoisotopic mass of neutral peptide Mr(calc): 1894.78. Ions score: 80

**Table S5.** Theoretical fragmentation of a peptide NVTVTHAQDILEK. Ions identified in the experimental MS/MS spectrum by the Mascot software are marked red.

| **#** | **a** | **a^++^** | **b** | **b^++^** | **b*** | **b*^++^** | **b^0^** | **b^0++^** | **Seq.** | **y** | **y^++^** | **y*** | **y*^++^** | **y^0^** | **y^0++^** | **#** |
| --- | --- | --- | --- | --- | --- | --- | --- | --- | --- | --- | --- | --- | --- | --- | --- | --- |
| **1** | **290.1347** | 145.5710 | 318.1296 | 159.5684 | 301.1030 | 151.0552 |  |  | **N** |  |  |  |  |  |  | **13** |
| **2** | **389.2031** | 195.1052 | **417.1980** | 209.1026 | 400.1714 | 200.5894 |  |  | **V** | ***1353.7373*** | 677.3723 | 1336.7107 | 668.8590 | 1335.7267 | 668.3670 | **12** |
| **3** | 490.2508 | 245.6290 | **518.2457** | 259.6265 | 501.2191 | 251.1132 | 500.2351 | 250.6212 | **T** | ***1254.6688*** | 627.8381 | 1237.6423 | 619.3248 | 1236.6583 | 618.8328 | **11** |
| **4** | 589.3192 | 295.1632 | **617.3141** | 309.1607 | 600.2875 | 300.6474 | 599.3035 | 300.1554 | **V** | ***1153.6212*** | 577.3142 | 1136.5946 | 568.8009 | 1135.6106 | 568.3089 | **10** |
| **5** | 690.3668 | 345.6871 | 718.3618 | 359.6845 | 701.3352 | 351.1712 | 700.3512 | 350.6792 | **T** | ***1054.5528*** | 527.7800 | 1037.5262 | 519.2667 | 1036.5422 | 518.7747 | **9** |
| **6** | 827.4258 | 414.2165 | 855.4207 | 428.2140 | 838.3941 | 419.7007 | 837.4101 | 419.2087 | **H** | ***953.5051*** | 477.2562 | 936.4785 | 468.7429 | 935.4945 | 468.2509 | **8** |
| **7** | 898.4629 | 449.7351 | 926.4578 | 463.7325 | 909.4312 | 455.2193 | 908.4472 | 454.7272 | **A** | ***816.4462*** | 408.7267 | 799.4196 | 400.2134 | 798.4356 | 399.7214 | **7** |
| **8** | 1026.5215 | 513.7644 | **1054.5164** | 527.7618 | 1037.4898 | 519.2485 | 1036.5058 | 518.7565 | **Q** | ***745.4090*** | 373.2082 | 728.3825 | 364.6949 | 727.3985 | 364.2029 | **6** |
| **9** | 1141.5484 | 571.2778 | **1169.5433** | 585.2753 | 1152.5168 | 576.7620 | 1151.5327 | 576.2700 | **D** | ***617.3505*** | 309.1789 | 600.3239 | 300.6656 | 599.3399 | 300.1736 | **5** |
| **10** | **1254.6325** | 627.8199 | 1282.6274 | 641.8173 | 1265.6008 | 633.3041 | 1264.6168 | 632.8120 | **I** | ***502.3235*** | 251.6654 | 485.2970 | 243.1521 | 484.3130 | 242.6601 | **4** |
| **11** | 1367.7165 | 684.3619 | 1395.7114 | 698.3594 | 1378.6849 | 689.8461 | 1377.7009 | 689.3541 | **L** | ***389.2395*** | 195.1234 | 372.2129 | 186.6101 | 371.2289 | 186.1181 | **3** |
| **12** | 1496.7591 | 748.8832 | 1524.7540 | 762.8807 | 1507.7275 | 754.3674 | 1506.7435 | 753.8754 | **E** | ***276.1554*** | 138.5813 | 259.1288 | 130.0681 | 258.1448 | 129.5761 | **2** |
| **13** |  |  |  |  |  |  |  |  | **K** | ***147.1128*** | 74.0600 | 130.0863 | 65.5468 |  |  | **1** |


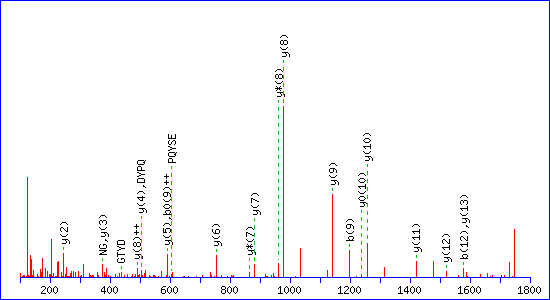


**Figure S6**. **MS/MS Fragmentation of NGTYDYPQYSEEAR.**
Monoisotopic mass of neutral peptide Mr(calc): 1894.78. Ions score: 80

**Table S6**. Theoretical fragmentation of a peptide NGTYDYPQYSEEAR. Ions identified in the experimental MS/MS spectrum by the Mascot software are marked red.

| **#** | **a** | **a^++^** | **b** | **b^++^** | **b*** | **b*^++^** | **b^0^** | **b^0++^** | **Seq.** | **y** | **y^++^** | **y*** | **y*^++^** | **y^0^** | **y^0++^** | **#** |
| --- | --- | --- | --- | --- | --- | --- | --- | --- | --- | --- | --- | --- | --- | --- | --- | --- |
| **1** | 30.0338 | 15.5206 | 58.0287 | 29.5180 |  |  |  |  | **G** |  |  |  |  |  |  | **15** |
| **2** | 347.1561 | 174.0817 | **375.1510** | 188.0792 | 358.1245 | 179.5659 |  |  | **N** | 1895.7930 | 948.4001 | 1878.7664 | 939.8869 | 1877.7824 | 939.3949 | **14** |
| **3** | 404.1776 | 202.5924 | 432.1725 | 216.5899 | 415.1460 | 208.0766 |  |  | **G** | ***1578.6707*** | 789.8390 | 1561.6441 | 781.3257 | 1560.6601 | 780.8337 | **13** |
| **4** | 505.2253 | 253.1163 | 533.2202 | 267.1137 | 516.1936 | 258.6005 | 515.2096 | 258.1084 | **T** | ***1521.6492*** | 761.3283 | 1504.6227 | 752.8150 | 1503.6387 | 752.3230 | **12** |
| **5** | 668.2886 | 334.6479 | 696.2835 | 348.6454 | 679.2570 | 340.1321 | 678.2729 | 339.6401 | **Y** | ***1420.6016*** | 710.8044 | 1403.5750 | 702.2911 | 1402.5910 | 701.7991 | **11** |
| **6** | 783.3155 | 392.1614 | 811.3105 | 406.1589 | 794.2839 | 397.6456 | 793.2999 | 397.1536 | **D** | ***1257.5382*** | 629.2727 | 1240.5117 | 620.7595 | 1239.5277 | 620.2675 | **10** |
| **7** | 946.3789 | 473.6931 | 974.3738 | 487.6905 | 957.3472 | 479.1773 | 956.3632 | 478.6852 | **Y** | ***1142.5113*** | 571.7593 | 1125.4847 | 563.2460 | 1124.5007 | 562.7540 | **9** |
| **8** | 1043.4316 | 522.2195 | 1071.4265 | 536.2169 | 1054.4000 | 527.7036 | 1053.4160 | 527.2116 | **P** | ***979.4480*** | 490.2276 | 962.4214 | 481.7143 | 961.4374 | 481.2223 | **8** |
| **9** | 1171.4902 | 586.2487 | **1199.4851** | 600.2462 | 1182.4586 | 591.7329 | 1181.4746 | 591.2409 | **Q** | ***882.3952*** | 441.7012 | 865.3686 | 433.1880 | 864.3846 | 432.6959 | **7** |
| **10** | 1334.5535 | 667.7804 | 1362.5485 | 681.7779 | 1345.5219 | 673.2646 | 1344.5379 | 672.7726 | **Y** | ***754.3366*** | 377.6719 | 737.3101 | 369.1587 | 736.3260 | 368.6667 | **6** |
| **11** | 1421.5856 | 711.2964 | 1449.5805 | 725.2939 | 1432.5539 | 716.7806 | 1431.5699 | 716.2886 | **S** | ***591.2733*** | 296.1403 | 574.2467 | 287.6270 | 573.2627 | 287.1350 | **5** |
| **12** | 1550.6282 | 775.8177 | **1578.6231** | 789.8152 | 1561.5965 | 781.3019 | 1560.6125 | 780.8099 | **E** | ***504.2413*** | 252.6243 | 487.2147 | 244.1110 | 486.2307 | 243.6190 | **4** |
| **13** | 1679.6708 | 840.3390 | 1707.6657 | 854.3365 | 1690.6391 | 845.8232 | 1689.6551 | 845.3312 | **E** | ***375.1987*** | 188.1030 | 358.1721 | 179.5897 | 357.1881 | 179.0977 | **3** |
| **14** | 1750.7079 | 875.8576 | 1778.7028 | 889.8550 | 1761.6762 | 881.3418 | 1760.6922 | 880.8497 | **A** | ***246.1561*** | 123.5817 | 229.1295 | 115.0684 |  |  | **2** |
| **15** |  |  |  |  |  |  |  |  | **R** | 175.1190 | 88.0631 | 158.0924 | 79.5498 |  |  | **1** |


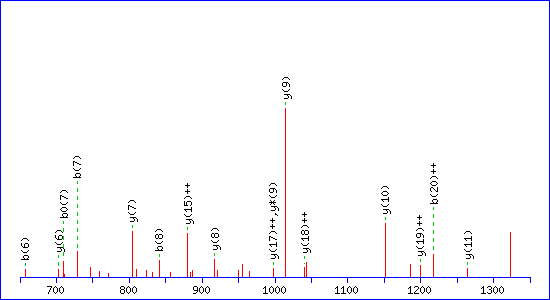


**Figure S7**. **MS/MS Fragmentation of CQTPIGAINSSMPFHNIHPLTIGECPK.**
Monoisotopic mass of neutral peptide Mr(calc): 3221.53. Ions score: 65

**Table S7.** Theoretical fragmentation of a peptide CQTPIGAINSSMPFHNIHPLTIGECPK. Ions identified in the experimental MS/MS spectrum by the Mascot software are marked red.

| **#** | **a** | **a^++^** | **b** | **b^++^** | **b*** | **b*^++^** | **b^0^** | **b^0++^** | **Seq.** | **y** | **y^++^** | **y*** | **y*^++^** | **y^0^** | **y^0++^** | **#** |
| --- | --- | --- | --- | --- | --- | --- | --- | --- | --- | --- | --- | --- | --- | --- | --- | --- |
| **1** | 133.0430 | 67.0251 | 161.0379 | 81.0226 |  |  |  |  | **C** |  |  |  |  |  |  | **27** |
| **2** | 261.1016 | 131.0544 | ***289.0965*** | 145.0519 | 272.0700 | 136.5386 |  |  | **Q** | 3078.5023 | 1539.7548 | 3061.4758 | 1531.2415 | 3060.4918 | 1530.7495 | **26** |
| **3** | 362.1493 | 181.5783 | ***390.1442*** | 195.5757 | 373.1176 | 187.0625 | 372.1336 | 186.5704 | **T** | 2950.4437 | 1475.7255 | 2933.4172 | 1467.2122 | 2932.4332 | 1466.7202 | **25** |
| **4** | 459.2020 | 230.1047 | 487.1969 | **244.1021** | 470.1704 | 235.5888 | 469.1864 | 235.0968 | **P** | 2849.3961 | 1425.2017 | 2832.3695 | 1416.6884 | 2831.3855 | 1416.1964 | **24** |
| **5** | 572.2861 | 286.6467 | ***600.2810*** | 300.6441 | 583.2545 | 292.1309 | 582.2704 | 291.6389 | **I** | 2752.3433 | 1376.6753 | 2735.3168 | 1368.1620 | 2734.3327 | 1367.6700 | **23** |
| **6** | 629.3076 | 315.1574 | ***657.3025*** | 329.1549 | 640.2759 | 320.6416 | 639.2919 | 320.1496 | **G** | 2639.2592 | 1320.1333 | 2622.2327 | 1311.6200 | 2621.2487 | 1311.1280 | **22** |
| **7** | 700.3447 | 350.6760 | ***728.3396*** | 364.6734 | 711.3130 | 356.1602 | 710.3290 | 355.6681 | **A** | 2582.2378 | 1291.6225 | 2565.2112 | 1283.1092 | 2564.2272 | 1282.6172 | **21** |
| **8** | 813.4287 | 407.2180 | ***841.4237*** | 421.2155 | 824.3971 | 412.7022 | 823.4131 | 412.2102 | **I** | 2511.2007 | 1256.1040 | 2494.1741 | 1247.5907 | 2493.1901 | 1247.0987 | **20** |
| **9** | 1130.5510 | 565.7792 | 1158.5460 | 579.7766 | 1141.5194 | 571.2633 | 1140.5354 | 570.7713 | **N** | 2398.1166 | 1199.5619 | 2381.0900 | 1191.0487 | 2380.1060 | 1190.5567 | **19** |
| **10** | 1217.5831 | 609.2952 | 1245.5780 | 623.2926 | 1228.5514 | 614.7794 | 1227.5674 | 614.2873 | **S** | 2080.9943 | 1041.0008 | 2063.9677 | 1032.4875 | 2062.9837 | 1031.9955 | **18** |
| **11** | 1304.6151 | 652.8112 | 1332.6100 | 666.8086 | 1315.5835 | 658.2954 | 1314.5994 | 657.8034 | **S** | 1993.9623 | 997.4848 | 1976.9357 | 988.9715 | 1975.9517 | 988.4795 | **17** |
| **12** | 1451.6505 | 726.3289 | 1479.6454 | 740.3263 | 1462.6189 | 731.8131 | 1461.6348 | 731.3211 | **M** | 1906.9302 | 953.9688 | 1889.9037 | 945.4555 | 1888.9197 | 944.9635 | **16** |
| **13** | 1548.7033 | 774.8553 | 1576.6982 | 788.8527 | 1559.6716 | 780.3394 | 1558.6876 | 779.8474 | **P** | 1759.8948 | 880.4511 | 1742.8683 | 871.9378 | 1741.8843 | 871.4458 | **15** |
| **14** | 1695.7717 | 848.3895 | 1723.7666 | 862.3869 | 1706.7400 | 853.8737 | 1705.7560 | 853.3816 | **F** | 1662.8421 | 831.9247 | 1645.8155 | 823.4114 | 1644.8315 | 822.9194 | **14** |
| **15** | 1832.8306 | 916.9189 | 1860.8255 | 930.9164 | 1843.7989 | 922.4031 | 1842.8149 | 921.9111 | **H** | 1515.7737 | 758.3905 | 1498.7471 | 749.8772 | 1497.7631 | 749.3852 | **13** |
| **16** | 1946.8735 | 973.9404 | 1974.8684 | 987.9379 | 1957.8419 | 979.4246 | 1956.8579 | 978.9326 | **N** | 1378.7147 | 689.8610 | 1361.6882 | 681.3477 | 1360.7042 | 680.8557 | **12** |
| **17** | 2059.9576 | 1030.4824 | 2087.9525 | 1044.4799 | 2070.9259 | 1035.9666 | 2069.9419 | 1035.4746 | **I** | ***1264.6718*** | 632.8395 | 1247.6453 | 624.3263 | 1246.6613 | 623.8343 | **11** |
| **18** | 2197.0165 | 1099.0119 | 2225.0114 | 1113.0093 | 2207.9849 | 1104.4961 | 2207.0008 | 1104.0041 | **H** | ***1151.5878*** | 576.2975 | 1134.5612 | 567.7842 | 1133.5772 | 567.2922 | **10** |
| **19** | 2294.0693 | 1147.5383 | 2322.0642 | 1161.5357 | 2305.0376 | 1153.0224 | 2304.0536 | 1152.5304 | **P** | ***1014.5288*** | 507.7681 | 997.5023 | 499.2548 | 996.5183 | 498.7628 | **9** |
| **20** | 2407.1533 | 1204.0803 | 2435.1482 | **1218.0778** | 2418.1217 | 1209.5645 | 2417.1377 | 1209.0725 | **L** | ***917.4761*** | 459.2417 | 900.4495 | 450.7284 | 899.4655 | 450.2364 | **8** |
| **21** | 2508.2010 | 1254.6041 | 2536.1959 | 1268.6016 | 2519.1694 | 1260.0883 | 2518.1853 | 1259.5963 | **T** | ***804.3920*** | 402.6996 | 787.3655 | 394.1864 | 786.3815 | 393.6944 | **7** |
| **22** | 2621.2851 | 1311.1462 | 2649.2800 | 1325.1436 | 2632.2534 | 1316.6303 | 2631.2694 | 1316.1383 | **I** | ***703.3443*** | 352.1758 | 686.3178 | 343.6625 | 685.3338 | 343.1705 | **6** |
| **23** | 2678.3065 | 1339.6569 | 2706.3014 | 1353.6544 | 2689.2749 | 1345.1411 | 2688.2909 | 1344.6491 | **G** | ***590.2603*** | 295.6338 | 573.2337 | 287.1205 | 572.2497 | 286.6285 | **5** |
| **24** | 2807.3491 | 1404.1782 | 2835.3440 | 1418.1757 | 2818.3175 | 1409.6624 | 2817.3335 | 1409.1704 | **E** | 533.2388 | 267.1230 | 516.2123 | 258.6098 | 515.2282 | 258.1178 | **4** |
| **25** | 2967.3798 | 1484.1935 | 2995.3747 | 1498.1910 | 2978.3481 | 1489.6777 | 2977.3641 | 1489.1857 | **C** | 404.1962 | 202.6017 | 387.1697 | 194.0885 |  |  | **3** |
| **26** | 3064.4325 | 1532.7199 | 3092.4274 | 1546.7174 | 3075.4009 | 1538.2041 | 3074.4169 | 1537.7121 | **P** | ***244.1656*** | 122.5864 | 227.1390 | 114.0731 |  |  | **2** |
| **27** |  |  |  |  |  |  |  |  | **K** | 147.1128 | 74.0600 | 130.0863 | 65.5468 |  |  | **1** |

**Figure S8.** **MALDI mass spectrum of the H5_hm_ antigen.**
The m/z values of the peaks are indicated. The H5_hm_ monomer as singly charged ion (m/z=69063), doubly charged ion (m/z=34199), and triply charged ion (m/z=22876), peak of the H5_hm_ dimer as singly charged ion (m/z=137420), peak of the H5_hm_ trimer as doubly charged ion (m/z=102834) were seen in the spectra.


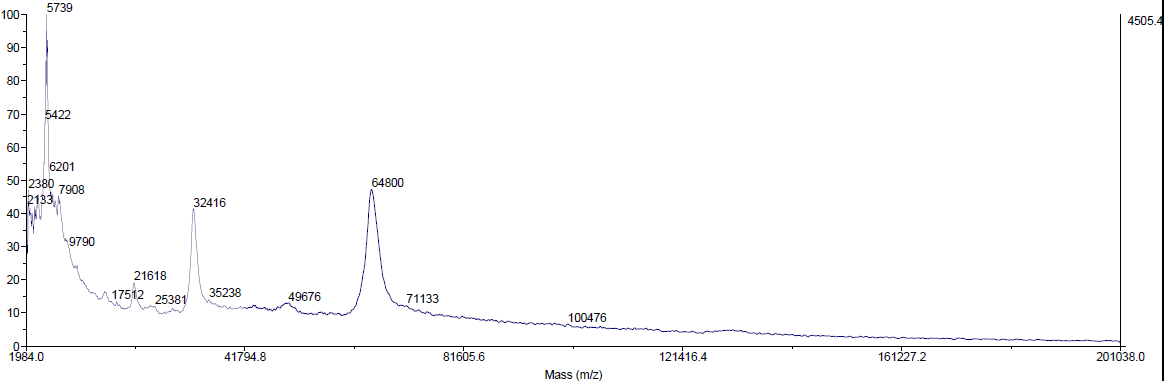


**Figure S9**. **MALDI mass spectrum of the H5_Man5_ antigen.**
The m/z values of the peaks are indicated. Peak of the H5_Man5_ monomer as singly charged ion (m/z=64800) and doubly charged ion (m/z=32416) was seen in the spectra.
